# A novel fluorescent probe-based flow cytometric assay for mineral-containing nanoparticles in serum

**DOI:** 10.1038/s41598-017-05474-y

**Published:** 2017-07-18

**Authors:** Edward R. Smith, Tim D. Hewitson, Michael M. X. Cai, Parisa Aghagolzadeh, Matthias Bachtler, Andreas Pasch, Stephen G. Holt

**Affiliations:** 1 Department of Nephrology, The Royal Melbourne Hospital, Melbourne, Victoria Australia; 2 Department of Medicine - Royal Melbourne Hospital, University of Melbourne, Melbourne, Victoria Australia; 3 Department of Clinical Research, University of Bern, Bern, Switzerland

## Abstract

Calciprotein particles, nanoscale aggregates of insoluble mineral and binding proteins, have emerged as potential mediators of phosphate toxicity in patients with Chronic Kidney Disease. Although existing immunochemical methods for their detection have provided compelling data, these approaches are indirect, lack specificity and are subject to a number of other technical and theoretical shortcomings. Here we have developed a rapid homogeneous fluorescent probe-based flow cytometric method for the detection and quantitation of individual mineral-containing nanoparticles in human and animal serum. This method allows the discrimination of membrane-bound from membrane-free particles and different mineral phases (amorphous vs. crystalline). Critically, the method has been optimised for use on a conventional instrument, without the need for manual hardware adjustments. Using this method, we demonstrate a consistency in findings across studies of Chronic Kidney Disease patients and commonly used uraemic animal models. These studies demonstrate that renal dysfunction is associated with the ripening of calciprotein particles to the crystalline state and reveal bone metabolism and dietary mineral as important modulators of circulating levels. Flow cytometric analysis of calciprotein particles may enhance our understanding of mineral handling in kidney disease and provide a novel indicator of therapeutic efficacy for interventions targeting Chronic Kidney Disease-Mineral Bone Disorder.

## Introduction

The plasma concentrations of calcium and phosphate required to generate and maintain our apatite-based endoskeleton are close to that which would precipitate in aqueous solution^1^. Thus, powerful inhibitory networks have evolved to prevent unwanted mineralisation outside of bone. In response to fluctuations in the concentration or flux of mineral ions, these homeostatic processes can bind and sequester excess mineral, delaying crystallisation and permitting safe disposal and clearance^2^. These defenses appear compromised in patients with Chronic Kidney Disease (CKD) and other metabolic diseases of accelerated aging, where they are either down-regulated or consumed, resulting in an increased propensity for extraosseous calcification^3^. Calciprotein particles (CPP) are nanoscale aggregates of insoluble mineral and various mineral-binding proteins like fetuin-A (Fet-A), and serve as one such mechanism to buffer disturbances in calcium and phosphate^4, 5^. Chronic dysregulation of mineral metabolism as seen in CKD, may result in the accumulation and ripening of these particles from the amorphous (CPP-I) to crystalline (CPP-II) state, resulting in toxicity^6^.

Serum CPP levels are elevated in CKD^7–9^, and are positively associated with serum phosphate, inflammatory and bone turnover markers^8^, coronary artery calcification^9^, aortic stiffness^8^ and mortality^10^. *In vivo*, CPP have been temporally implicated in the development of vascular calcification since they appear in the circulation of adenine-fed animals before detectable aortic mineral deposition^11^. This is in keeping with studies showing that mineral nanocrystals, rather than phosphate *per se*, drive vascular smooth muscle cell mineralisation *in vitro* ^12–14^. Administration of bisphosphonate, a crystal poison, has also been shown to ameliorate vascular calcification^11^ and the nephrotoxic effects of phosphate loading in rats^15^. Based on this data, we^6^ and others^16^ have theorised that CPP may be potential mediators of cardiovascular and renal ‘phosphotoxicity’ in CKD, providing the ‘missing link’ that connects disturbances in mineral metabolism with downstream effects, even when circulating phosphate levels remain apparently ‘normal’.

Fet-A is a ~58 kDa liver-derived mineral-binding glycoprotein abundant in serum and the principal protein component present in synthetic and endogenous CPP^17^. As a negative acute phase reactant^18^, levels are found to be suppressed in chronic inflammatory states like those with end-stage kidney disease^19, 20^ and rheumatic disease^7^. Consistent with its well-described role as a systemic regulator of extracellular matrix mineralisation^21, 22^, Fet-A deficiency has been associated with increased vascular calcification scores and risk of cardiovascular mortality in patients with advanced CKD^20, 23^.

Existing studies of CPP have relied on the immunochemical detection of Fet-A, in a high-molecular weight fraction after centrifugation (>16,000 *g*). Hamano *et al*. published the original indirect ELISA method for the quantitation of serum CPP bound Fet-A^9^, which was based on the difference between the concentration of Fet-A in serum (free Fet-A + CPP Fet-A) and after sedimentation and removal of CPP from the same sample with high-speed centrifugation (free Fet-A). Although we have used this method extensively (after minor modifications)^8^ and it performs reproducibly in our hands^7, 8, 10, 24, 25^ and others^9, 26^, several theoretical and technical shortcomings were apparent^27^. These relate to: (1) the use of fetuin-A as a surrogate for mineral-containing particles given its presence in various particulates that may also be sedimented with CPP (apoptotic bodies^28^, exosomes^29^, opsonised cell debris^30, 31^); (2) failure to detect fetuin-A-poor CPP in states of fetuin-A deficiency (i.e. patients with end-stage renal disease^19^); (3) substantial analytical error encountered when deriving estimates of CPP Fet-A levels based on the small numerical differences between total fetuin-A in serum and after centrifugation following substantial dilution of each (1 in 10,000); (4) that measurements provide a readout of fetuin-A protein enrichment rather than particle number; and (5) that such measurements do not allow the discrimination of CPP-I from CPP-II, which may have profoundly different biological effects^32^. Thus, immunochemical measurements based on fetuin-A mass alone may not be specific for mineral-containing particles nor a reliable index of mineral content, and critically, do not permit proper enumeration or discriminative analyses of the circulating pool. This prompted us to look for alternative analytical strategies.

Flow cytometry is well suited to the multi-parameter characterisation and quantitation of particles, and has been applied extensively to the analysis of membrane-bound extracellular vesicles, and, in particular, exosomes (diameter ~30–150 nm)^33, 34^. Given their similar size distribution to these nanoparticles, we explored the possibility of using the same technology for quantitative CPP analysis. Here we describe a novel flow cytometric-based method using mineral- and membrane-specific fluorescent probes for the direct quantitation of individual CPP in serum. Further we illustrate how application of this new method provides insight into dysregulated mineral handling in CKD and the origin of these particles *in vivo*.

## Results

### Antibody-based labelling strategy for CPP

Initially, we developed an antibody labelling strategy for CPP based on previously published proteomic analyses^9, 35^, staining for two protein targets ubiquitously present in CPP: fetuin-A (FetA) and apolipoprotein A1 (ApoA1). However, while CPP could be stained for both these proteins (see Supplementary Fig. S2), they also stained positively with a panel of antibodies against unrelated antigens not present in CPP, alternate subclass IgG controls and non-human species-specific targets (see Supplementary Fig. S2). This is consistent with previous reports of non-specific adsorption of various antibodies to mineral microparticles and tissue deposits^36, 37^. We therefore considered antibody-based detection of CPP unreliable and prone to false positives due to non-specific binding and adsorption of immunoglobulin. Indeed, IgG and IgM have previously been detected in proteomic studies of these particle fractions^9, 35^. We therefore decided to pursue non-immunochemical means of staining using low molecular weight fluorescent probes. Background information regarding the use of light scattering and fluorescence parameters in the study of nanoparticles can be found in the Supplementary material.

### Application of mineral- and membrane-specific fluorescent probes to the analysis of CPP

**Figure 1:**
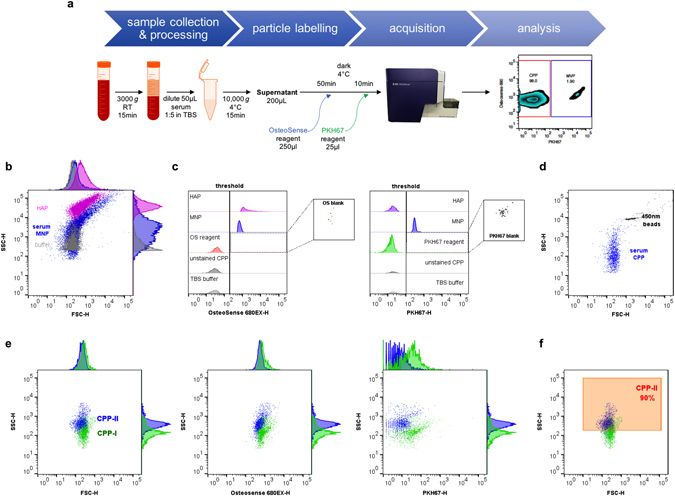
Flow cytometric detection of mineral-containing nanoparticles by fluorescence-based thresholding. (**a**) Schematic of method through pre-analytical and analytical phases, (**b**) Dot plot showing the overlap of endogenous serum mineral-containing nanoparticles (blue) and buffer (grey) by light scatter with minimum SSC threshold applied. Hydroxyapatite nanoparticles (<200 nm; purple) included as comparator. (**c**) Histograms showing fluorescence thresholds for OsteoSense 680EX and PKH67 based on respective reagent blanks and unstained particles. Background counts with thresholds applied shown in adjacent plots. (**d**) Representative dot plot showing scatter characteristics of endogenous CPP subpopulation (OsteoSense+ve/PKH67−ve) and red fluorescent 450 nm beads. (**e**) Dot plots with adjunct histograms showing the partial separation of OsteoSense 680-labelled human serum-derived CPP-I (amorphous; green) and CPP-II (crystalline; blue) using SSC, but not OsteoSense680 staining intensity, with fluorescence threshold trigger. Note, CPP do not stain for PKH67. (**f**) Dot plot showing the position of the CPP-II gate (orange), based on the 90^th^ centile by SSC. CPP, calciprotein; HAP, hydroxyapatite; MNP, mineral-containing nanoparticles, OS, OsteoSense; TBS, Tris-buffered saline.

Bisphosphonates are pyrophosphate analogues with exquisite affinity (*K*_*L*_ > 1 × 10^6^ L mol^−1^) and specificity for mineral phases^38^. Accordingly, fluorescently-conjugated bisphosphonate derivatives have already been extensively applied to *in vivo* imaging studies of bone^39^ and ectopic calcification^40–42^, as well as flow cytometric-based assays of mineral-containing exosomes isolated from culture media and murine serum^43^. Here we utilise one such fluorescent derivative, OsteoSense 680EX. Since mineral deposits have also been shown to reside within, or seed from the surface of, microvesicular structures - distinct from membrane-free CPP - we further enhanced analytical specificity by the addition of a green fluorescent membrane intercalating dye, PKH67. A double staining protocol using OsteoSense 680EX and PKH67 was developed (Fig. 1a).

### Gating strategy and thresholding

We set sizing gates empirically, positioning 500 nm latex beads on the fifth decade of side scatter-height (SSC-H), which is well within the acquisition window for the detector (linearity maximum <2.2 × 10^5^). Detection of OsteoSense 680EX positive events was triggered from the 640 nm red laser using the APC-Cy7 detector. On theoretical grounds, excitation using a lower wavelength (violet or blue) laser would increase light scattering and maximize excitation of fluorochromes and the detection sensitivity of individual particle detection (although may also incur substantial photobleaching effects). However, alternative commercially-available mineral-binding dyes with lower excitation wavelengths show substantial spectral overlap with PKH67. We therefore selected the derivative with the lowest excitation wavelength (680 nm maxima) but non-overlapping spectra.

Expectedly, the scatter profile of endogenous mineral-containing nanoparticles (MNP) overlaps substantially with that generated by the buffer and electronics (Fig. 1b). For comparison, we also ran a suspension of hydroxyapatite nanoparticles (HAP), which despite their small size (<200 nm; see Supplementary Fig. S4), have very high SSC intensities. Detection thresholds were determined empirically, based on the fluorescence of buffer/reagent blanks and unstained particles (Fig. 1c). Applying this threshold, event rates for reagent blanks were consistently <20 events/min. Lowering of the detection threshold did not appreciably increase the number of particles detected. However, we cannot exclude the possibility of missing very dimly stained particles. Lower thresholds can be applied with the use of an additional centrifugation step to remove unbound dye/dye aggregates, although this greatly impacted on recovery (15–52% of unspun levels for total CPP/μL) and increased overall imprecision due to system noise (within-run CV > 20% vs. <5% for total CPP/μL). Unstained particles appeared to have minimal autofluorescence above that of the buffer. With this setup, serum (endogenous) CPP could be detected and resolved from nonfluorescent noise and unbound dye, as a polydisperse population with SSC-H intensities less than that of 450 nm polystyrene beads (Fig. 1d). Such polydispersity is consistent with the morphological heterogeneity seen by us on imaging previously^6, 27, 44^. Next we applied the method to synthetically generated CPP-I and CPP-II. Both CPP-I and CPP-II stained with OsteoSense 680EX, but not PKH67, consistent with the presence of mineral and absence of a membrane (Fig. 1e). CPP-II showed more intense, but overlapping (<10%), scatter than CPP-I, consistent with size distribution analyses by other light scattering techniques and transmission electron microscopy (TEM) imaging^44, 45^. The intensity of OsteoSense 680EX fluorescence was very similar for CPP-I and CPP-II and therefore offers no additional discrimination. Batches of CPP-I and CPP-II were generated using multiple pools of human serum (n = 20) as previously described^45^, and used to define gates encompassing 90^th^ centile of all CPP-II particles (Fig. 1f). The lack of separation between CPP-I and CPP-II also undoubtedly reflects the inability of the system to fully discriminate small differences in particle size/composition. Therefore, CPP-II counts based on SSC-H gates only provide a measure of CPP-II abundance and not absolute numbers of crystalline particles.

### Discrimination of CPP and membrane-bound particles

We confirmed the specificity of OsteoSense 680EX staining for mineral by incubating CPP with ethylenediaminetetraacetic acid (EDTA; a mineral chelator), and monitoring the dissolution of particles over 24 hours (see Supplementary Fig. S3A). Similarly, specificity of PKH67 for membrane was demonstrated by incubating CPP with detergent (Triton X-100), resulting in the solubilisation of only the membrane-bound particle (MBP) component (see Supplementary Fig. S3B). Since detergent action only dissolves away membrane and not mineral, MBP gates could not be defined based on this treatment, and lower bounds were instead set using unstained CPP and PKH67 reagent blank (Fig. 1E). Interestingly, MBP populations consistently showed more intense scatter than CPP (presumably due to differences in optical properties given their equivalent size). TEM analysis of particles pelleted using a stepped centrifugation protocol confirmed the presence of CPP, and membrane-bound mineral-containing vesicles at higher speeds (see Supplementary Fig. S4). Importantly, vesicular structures were seldom present in pellets spun at 30,000 *g* for 2 h (e.g. see Supplementary Fig. S7), which we routinely use to prepare CPP from serum *in vitro*. Of note, while HAP nanoparticles stained strongly for OsteoSense 680EX, they remained PKH67 negative (Fig. 1c). As a final test of specificity, we demonstrated that OsteoSense 680EX -positive MNP could be sedimented using high-speed centrifugation (50,000 *g*, 2 h and 4 °C; see Supplementary Fig. S3C). Overall, the ability to discriminate CPP-I from CPP-II (albeit partially) and membrane-bound mineral-containing particles clearly provides an important step forward in analytical capability. Details regarding the quantitation of individual mineral-containing nanoparticles and comparison with measurements made by nanoparticle tracking analysis (NTA) and an alternative flow cytometer are provided in the Supplementary material.

### Flow cytometry of serum mineral-containing nanoparticles in a cohort of dialysis patients

**Figure 2:**
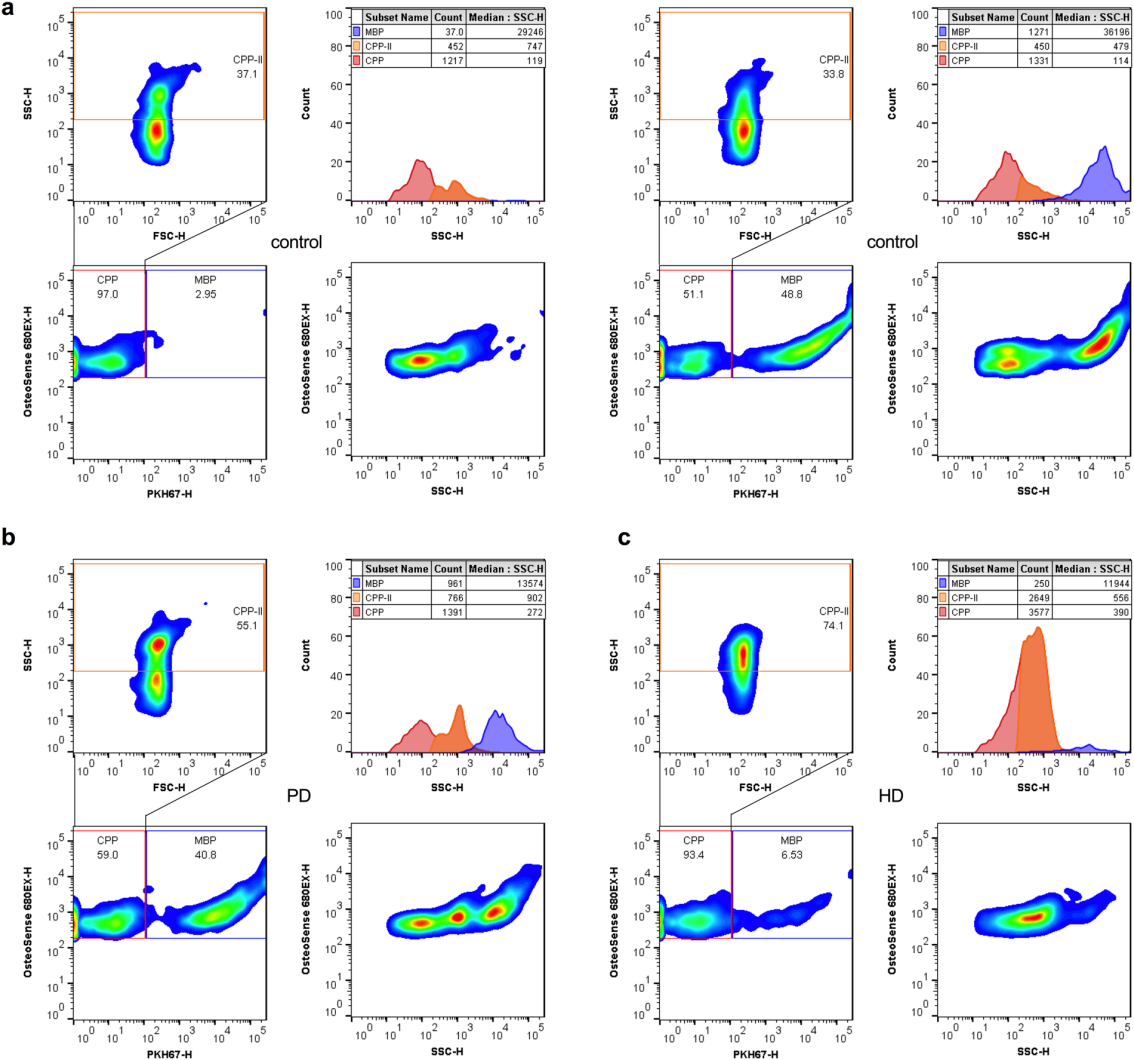
Flow cytometric analysis of mineral-containing nanoparticles in human serum. (**a**–**c**) representative cytograms showing dual OsteoSense680EX and PKH67 staining on mineral-containing nanoparticles in serum from (**a**) adult controls, (**b**) peritoneal dialysis patient and (**c**) haemodialysis patient. Gates and population percentiles for CPP (red), MBP (blue) and CPP-II (orange) as shown. Histograms show gated populations and their respective volumetric measurements (counts/μL). CPP, calciprotein particles; MBP, membrane-bound particles; HD, haemodialysis; PD, peritoneal dialysis.

To further validate the utility of the method, we analysed serum samples from 80 patients with end-stage renal disease undergoing peritoneal dialysis (PD; n = 40) or haemodialysis (HD; n = 40), who were recruited to an ongoing observational study at our centre^7, 25^. Samples from individuals without CKD were also obtained to serve as controls (n = 40). Representative readouts for mineral-containing particles in human serum obtained from control, PD and HD groups are shown in Fig. 2.

**Figure 3:**
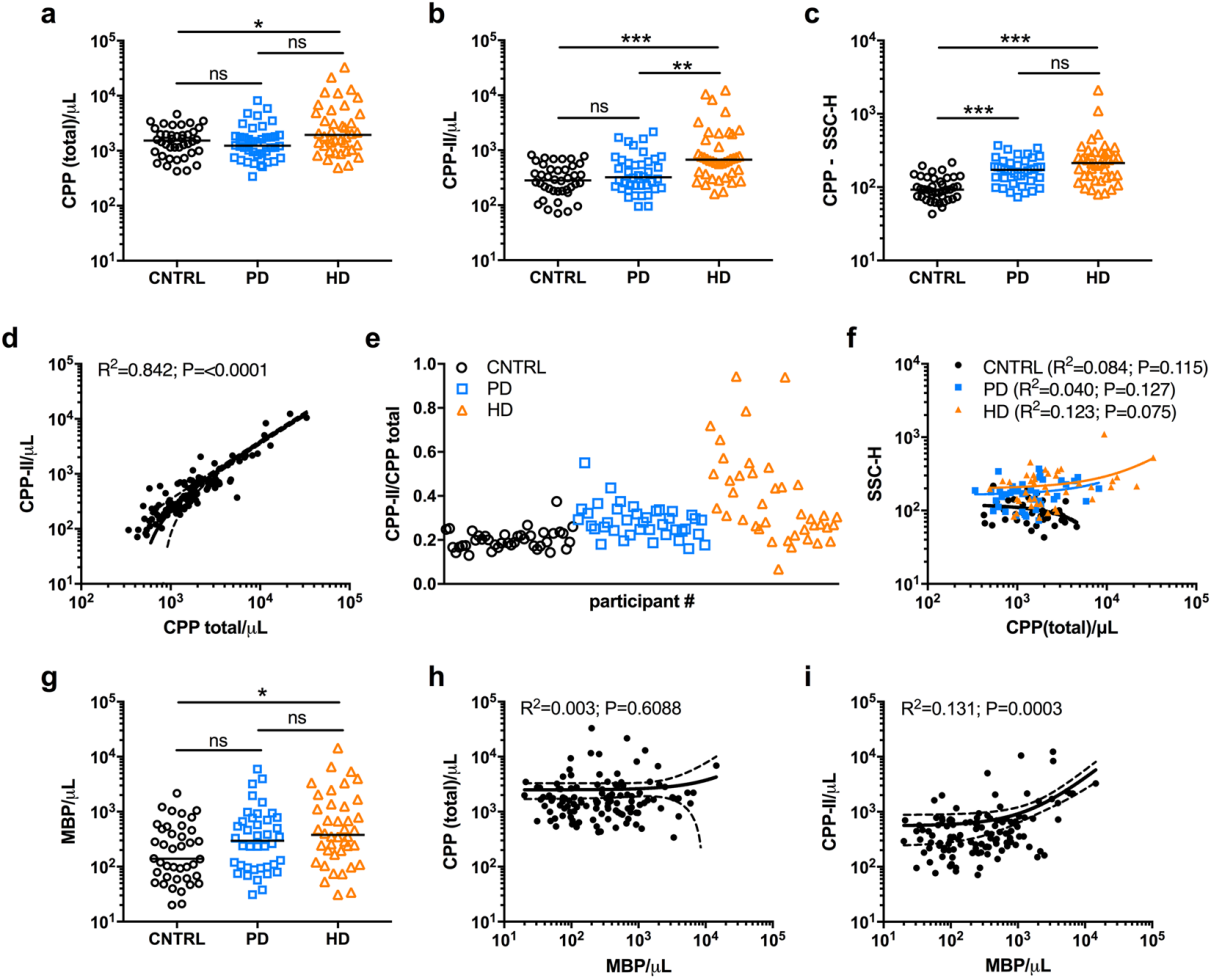
Uraemia is associated with the accumulation and ripening of CPP in patients with end-stage kidney disease. (**a**–**c**) Differences in (**a**) total CPP, (**b**) CPP-II and (**c**) SSC-H between control, PD and HD groups. (**d**) correlation of total CPP and CPP-II counts in all patients (n = 120). Line = median value. (**e**) Variability in the CPP-II/total CPP ratio between patient groups as for (**a**). (**f**) Lack of association between SSC-H and total CPP counts/μL in each group. (**g**) Between group differences in MBP and (**h**) the lack of association with total CPP counts. (**i**) Weak association of CPP-II and MBP counts. P-values for One-way ANOVA with Bonferroni correction or Kruskal-Wallis test with Dunns post-test for multiple comparisons (as appropriate) denoted as *P < 0.05, **P < 0.01, ***P < 0.001. For non-linear regression analyses (**d**,**h**,**i**), regression line (solid) with 95% confidence intervals (dashed) are shown. CNTRL, control; CPP, calciprotein particles; MBP, membrane-bound particles; HD, haemodialysis; PD, peritoneal dialysis.

Total serum CPP levels were modestly raised in the HD group compared to controls (Fig. 3a), whereas CPP-II levels were higher in the HD group compared to PD and control groups (Fig. 3b). SSC-H, on the other hand, was higher in PD and HD groups compared to controls (Fig. 3c). Overall, total CPP and CPP-II counts were strongly correlated (Fig. 3d), although CPP-II as a proportion of total CPP levels was generally greater and more variable in those on HD (Fig. 3e). Particle counts showed no relationship with SSC-H overall, or in any individual group (Fig. 3f). Again, MBP counts appeared modestly elevated in HD compared to control groups (Fig. 3g), but, while unrelated to total CPP counts (Fig. 3h), were weakly associated with CPP-II levelsin a non-linear fashion (Fig. 3i). Between group differences in serum inflammatory cytokines (interleukin (IL)-1β, IL-6 and tumour necrosis factor (TNF)-α) are depicted in Supplementary Figure S7 and, in keeping with published data^46^, were elevated in HD and PD groups compared to controls. As shown in Table 1, in pooled analysis of HD and PD patients, both total CPP and CPP-II counts were higher in diabetics, and correlated strongly with serum phosphate, PTH, intact FGF23, IL-1β, IL-6, sclerostin (an inhibitor of Wnt/β-catenin signalling) and weakly with osteoprotegerin (a soluble decoy receptor for RANKL). Given the co-correlation of total CPP and CPP-II it is expected that both measures would associate with the same variables, however, it should be noted that coefficients were consistently greater for CPP-II than for total CPP levels. CPP-II was also weakly correlated with serum calcium and TNF-α and inversely with serum magnesium and bicarbonate concentrations. SSC-H was associated with the same biochemistries as CPP-II. Regression analyses of CPP-II and inflammatory markers are shown in Supplementary Figure S7. Interestingly, MBP levels were only weakly correlated with TNF-α and IL-6. No parameters from this analysis were associated with age, gender, serum albumin, Dickkopf-related protein 1 (DKK-1; Wnt signaling inhibitor), osteocalcin or osteopontin (both mineral-binding proteins) or the presence of residual renal function, although the numbers with the latter were small (n = 8).

**Table 1:** Association of mineral nanoparticle flow cytometric parameters with demographic and biochemical measurements in a cohort of dialysis patients.

Variable (units)	n = 80*	CPP (total)		SSC-H		CPP-II		MBP	
		r (95% CI)	P-value	r (95% CI)	P-value	r (95% CI)	P-value	r (95% CI)	P-value
age (years)	61 ± 18	0.04 (−0.19 to 0.27)	0.7298	0.11 (−0.12 to 0.33)	0.3219	0.08 (−0.15 to 0.30)	0.4926	0.14 (−0.09 to 0.36)	0.2052
gender (male = 1)	57%	0.02 (−0.20 to 0.23)	0.8532	0.00 (−0.22 to 0.22)	0.9715	0.02 (−0.35 to 0.39)	0.9173	0.04 (−0.20 to 0.27)	0.7629
diabetic (yes = 1)	44%	**0**.**48** (**0**.**22 to 0**.**68**)	**0**.**0004**	**0**.**57** (**0**.**30 to 0**.**76**)	**0**.**0001**	**0**.**56** (**0**.**28 to 0**.**76**)	**0**.**0003**	0.32 (−0.01 to 0.61)	0.0517
serum total calcium (mmol/L)	2.22 ± 0.20	0.18 (−0.07 to 0.41)	0.1354	0.14 (−0.10 to 0.37)	0.2444	0.29 (0.06 to 0.49)	0.0134	0.16 (−0.08 to 0.39)	0.1725
serum phosphate (mmol/L)	1.59 ± 0.48	**0**.**40** (**0**.**27 to 0**.**58**)	<**0**.**0001**	**0**.**17** (**0**.**01 to 0**.**35**)	**0**.**0375**	**0**.**44** (**0**.**23 to 0**.**60**)	<**0**.**0001**	−0.13 (−0.31 to 0.06)	0.1687
serum magnesium (mmol/L)	1.10 ± 0.39	−0.25 (−0.50 to 0.04)	0.0852	**−0**.**31** (**−0**.**55 to −0**.**02**)	**0**.**0323**	**−0**.**30** (**−0**.**49 to −0**.**07**)	**0**.**0081**	0.11 (−0.11 to 0.33)	0.3152
serum bicarbonate (mmol/L)	22.5 ± 4.9	−0.05 (−0.26 to 0.16)	0.6334	**−0**.**22** (**−0**.**43 to −0**.**01**)	**0**.**0496**	**−0**.**21** (**−0**.**01 to −0**.**40**)	**0**.**0402**	−0.04 (−0.27 to 0.20)	0.7499
serum albumin (g/L)	35 ± 4	−0.11 (−0.34 to 0.13)	0.3414	−0.13 (−0.36 to 0.10)	0.2603	−0.20 (−0.41 to 0.03)	0.0830	0.14 (−0.10 to 0.35)	0.2457
plasma PTH (pmol/L)	24 (14–58)	**0**.**14** (**0**.**05 to 0**.**35**)	**0**.**0111**	**0**.**14** (**0**.**10 to 0**.**31**)	**0**.**0230**	**0**.**21** (**0**.**11 to 0**.**37**)	**0**.**0037**	−0.25 (−0.53 to 0.07)	0.1152
serum 25(OH) vitamin D† (nmol/L)	47 (38–72)	−0.14 (−0.37 to 0.11)	0.2568	**−0**.**32** (**−0**.**55 to −0**.**05**)	**0**.**0199**	0.05 (−0.25 to 0.34)	0.7412	0.04 (−0.26 to 0.34)	0.7811
serum intact FGF23 (pg/mL)	215 (42–980)	**0**.**39** (**0**.**23 to 0**.**55**)	<**0**.**0001**	**0**.**24** (**0**.**01 to 0**.**44**)	**0**.**0351**	**0**.**35** (**0**.**21 to 0**.**49**)	<**0**.**0001**	0.00 (−0.27 to 0.29)	0.9567
serum IL-1β (pg/mL)	27 (16–57)	**0**.**34** (**0**.**16 to 0**.**50**)	<**0**.**0001**	**0**.**29** (**0**.**08 to 0**.**47**)	**0**.**0060**	**0**.**43** (**0**.**25 to 0**.**56**)	<**0**.**0001**	0.03 (−0.21 to 0.27)	0.7837
serum TNF-α (pg/mL)	39 (26–53)	0.13 (−0.09 to 0.33)	0.2213	**0**.**23** (**0**.**01 to 0**.**42**)	**0**.**0313**	**0**.**23** (**0**.**02 to 0**.**42**)	**0**.**0291**	**0**.**24** (**0**.**02 to 0**.**44**)	**0**.**0301**
serum IL-6 (pg/mL)	9.2 (4.2–16.7)	**0**.**23** (**0**.**06 to 0**.**46**)	**0**.**0094**	**0**.**27** (**0**.**05 to 0**.**45**)	**0**.**0117**	**0**.**34** (**0**.**14 to 0**.**52**)	**0**.**0009**	**0**.**26** (**0**.**03 to 0**.**47**)	**0**.**0259**
serum DKK-1 (pg/mL)	2143 ± 898	0.11 (−0.12 to 0.33)	0.3181	−0.12 (−0.32 to 0.09)	0.2463	−0.07 (−0.27 to 0.14)	0.5169	0.05 (−0.18 to 0.28)	0.6667
serum OCN (pg/mL)	2951 (834–7560)	0.10 (−0.10 to 0.30)	0.2955	0.16 (−0.08 to 0.37)	0.1736	−0.10 (−0.23 to 0.18)	0.7761	−0.12 (−0.32 to 0.09)	0.5169
serum OPG (pg/mL)	825 (323–1755)	**0**.**25** (**0**.**05 to 0**.**42**)	**0**.**0114**	**0**.**19** (**−0**.**01 to 0**.**38**)	**0**.**0500**	**0**.**21** (**0**.**01 to 0**.**39**)	**0**.**0355**	0.15 (−0.05 to 0.34)	0.1293
serum OPN (pg/mL)	2566 (1730–8235)	−0.20 (−0.40 to 0.03)	0.0774	0.03 (−0.16 to 0.24)	0.6928	−0.17 (−0.39 to 0.07)	0.1588	0.10 (−0.10 to 0.29)	0.3042
serum sclerostin (pg/mL)	2055 ± 1024	**0**.**31** (**0**.**11 to 0**.**48**)	**0**.**0117**	**0**.**12** (**0**.**01 to 0**.**23**)	**0**.**0401**	**0**.**26** (**0**.**06 to 0**.**43**)	**0**.**0095**	−0.19 (−0.38 to 0.01)	0.0516

*Expressed as mean ± SD or median (25th–75th percentile); ^†^n = 71. Associations with P < 0.05 are given in bold text.

CPP, calciprotein particle; DKK-1, Dickkopf-related protein 1; FGF, fibroblast growth factor; IL, interleukin; MBP, membrane-bound particle;

OCN, osteocalcin; OPG, osteoprotegerin; OPN, osteoponitn; PTH, parathyroid hormone; SSC-H, side scatter - height; TNF, tumour necrosis factor.

In head-to-head analysis of the same samples, CPP Fet-A levels measured by ELISA were strongly associated with both CPP-II (r = 0.475, p = 0.0026) and SSC-H (r = 0.603, p = 0.0380) parameters by flow cytometry, demonstrating good agreement between methods.

### Flow cytometric analysis of calciprotein particles in adenine-treated uraemic rats and the response to dietary phosphate

An important advantage of the current method is that it circumvents issues relating to the use of species-specific antisera and thus facilitates experimental modeling in animals. Indeed, attempts to measure circulating CPP in animal models have until now been restricted to qualitative and semi-quantitative analyses using Western blotting^11, 47, 48^. CPP (also known as fetuin-mineral complexes) have been detected in the serum of adenine-treated (0.75%) rats in as little as two weeks following dietary supplementation, even in the absence of changes in serum phosphate concentration^11^. This implied that the emergence of circulating CPP might be an early manifestation of the derangements in mineral metabolism seen in CKD. While some features of the adenine model are not representative of CKD (e.g. polyuria, weight loss), it does nonetheless recapitulate many of the features of advanced human CKD-MBD (depending on the dose and duration of administration): hyperphosphataemia, secondary hyperparathyroidism, FGF23 excess, calcitriol deficiency, high turnover osteodystrophy and ectopic calcification.

**Figure 4:**
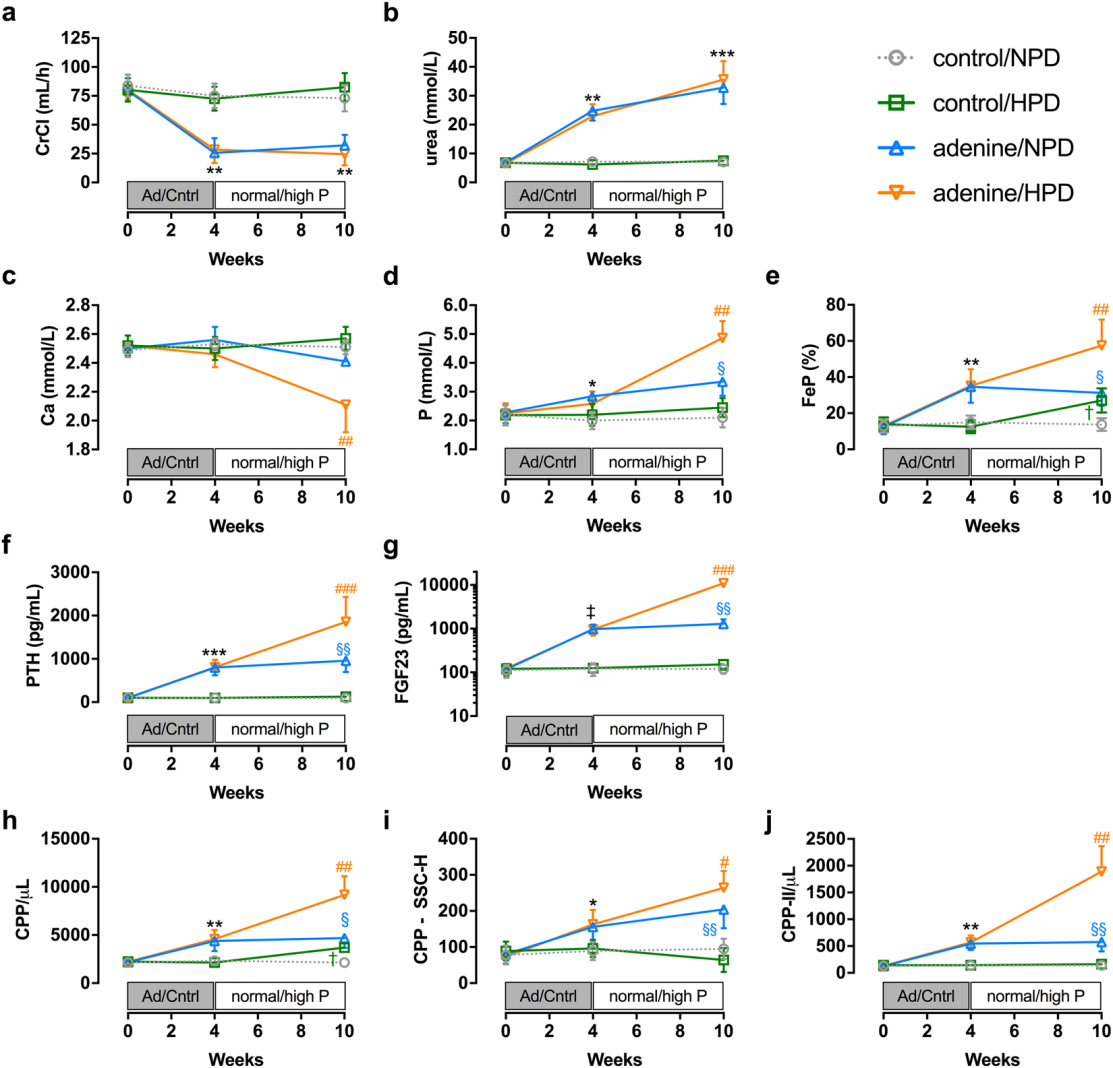
Adenine-induced renal failure in rats is associated with calciprotein ripening and is enhanced by a high phosphate diet. Changes in (**a**) CrCl, (**b**) serum urea, (**c**) serum Ca, (**d**) serum P, (**e**) urinary FeP, (**f**) serum intact PTH, (**g**) serum intact FGF23 and serum flow cytometric parameters (**h**) total CPP, (**i**) CPP SSC-H and (**j**) CPP-II levels in control and adenine-treated rats fed normal or high-phosphate containing diets over the 10-week study period. *P < 0.05, **P < 0.01, ***P < 0.001, ^‡^ *P* < 0.0001 for adenine-treated groups vs. control groups; ^#^ *P* < 0.05, ^##^ *P* < 0.01, ^*###*^ *P* ≤ 0.0001 for HPD-fed adenine-treated group vs. NPD-fed adenine-treated group; ^§^ *P* < 0.05, ^§§^ *P* < 0.01 for NPD-fed adenine-treated group vs. NPD-fed control group; ^†^ *P* < 0.05 for HPD-fed control group vs. NPD-fed control group. Data presented as mean ± SEM for each experimental group (n = 15 per group). Note logarithmic scale in (**g**). Ad, adenine; Ca, calcium; Cntrl, control; CPP, calciprotein particles; CrCl, creatinine clearance; FeP, fractional excretion of phosphate; FGF23, fibroblast growth factor 23; HPD, high phosphate diet; NPD, normal phosphate diet; P, phosphate; PTH, parathyroid hormone; SSC-H, side scatter-height.

In our hands, 4 weeks of dietary adenine supplementation in rats (0.75% for 2 weeks then 0.5% for 2 weeks, n = 30) resulted in the induction of uraemia (Fig. 4a,b) and elevations in phosphate (Fig. 4d), fractional phosphate excretion (FeP; Fig. 4e), intact PTH (Fig. 4f) and FGF23 (Fig. 4g), consistent with previous reports employing the same treatment protocol^49^. Total serum CPP levels were elevated in adenine-treated animals compared to baseline (week 0) and normal renal function controls (Fig. 4h; mean 56% increase), with CPP-II levels increased more than 4-fold from baseline (Fig. 4j). All parameters remained stable in the control animals fed standard chow. After the 4-week induction period, adenine-treated and control animals were further divided into two subgroups (n = 15/group; matched for body weight, creatinine and phosphate) and allocated to receive either standard chow (normal phosphate diet; NPD) or a high-phosphate diet (HPD) for a further 6 weeks. As expected, HPD feeding in adenine-treated animals resulted in marked elevations in phosphate (Fig. 4d), FeP (Fig. 4e), PTH (Fig. 4f) and FGF23 (Fig. 4e), as well as a reduction in serum calcium concentration (Fig. 4c) compared to NPD-fed adenine-treated animals by week 10. In HPD-fed adenine-treated rats these changes were accompanied by an increase in total CPP levels (Fig. 4h), and a striking increase in CPP-II levels by week 10 (>14-fold over baseline; Fig. 4j). TEM analysis of the CPP-containing fraction from pooled adenine-treated/HPD-fed rat serum confirmed the presence of particles with morphologies consistent with CPP-I and CPP-II synthesised from human serum *in vitro* (see Supplementary Fig. S8).

Dietary phosphate supplementation in control rats had no effect on conventional serum biochemical parameters over the study period but resulted in increased FeP at 10 weeks compared to baseline and normal phosphate-fed controls (Fig. 4e). We also observed an increase in total serum CPP levels in HPD-fed controls by week 10 (Fig. 4h), equivalent in magnitude to that seen in adenine-treated rats allocated the control diet. Since high-phosphate feeding in these control animals was associated with a slight reduction in mean SSC-H (i.e. smaller particle size and/or reduced crystallinity) this is likely to reflect increased formation of CPP-I (Fig. 4i).

### Flow cytometric analysis of calciprotein particles in other animal models

**Figure 5:**
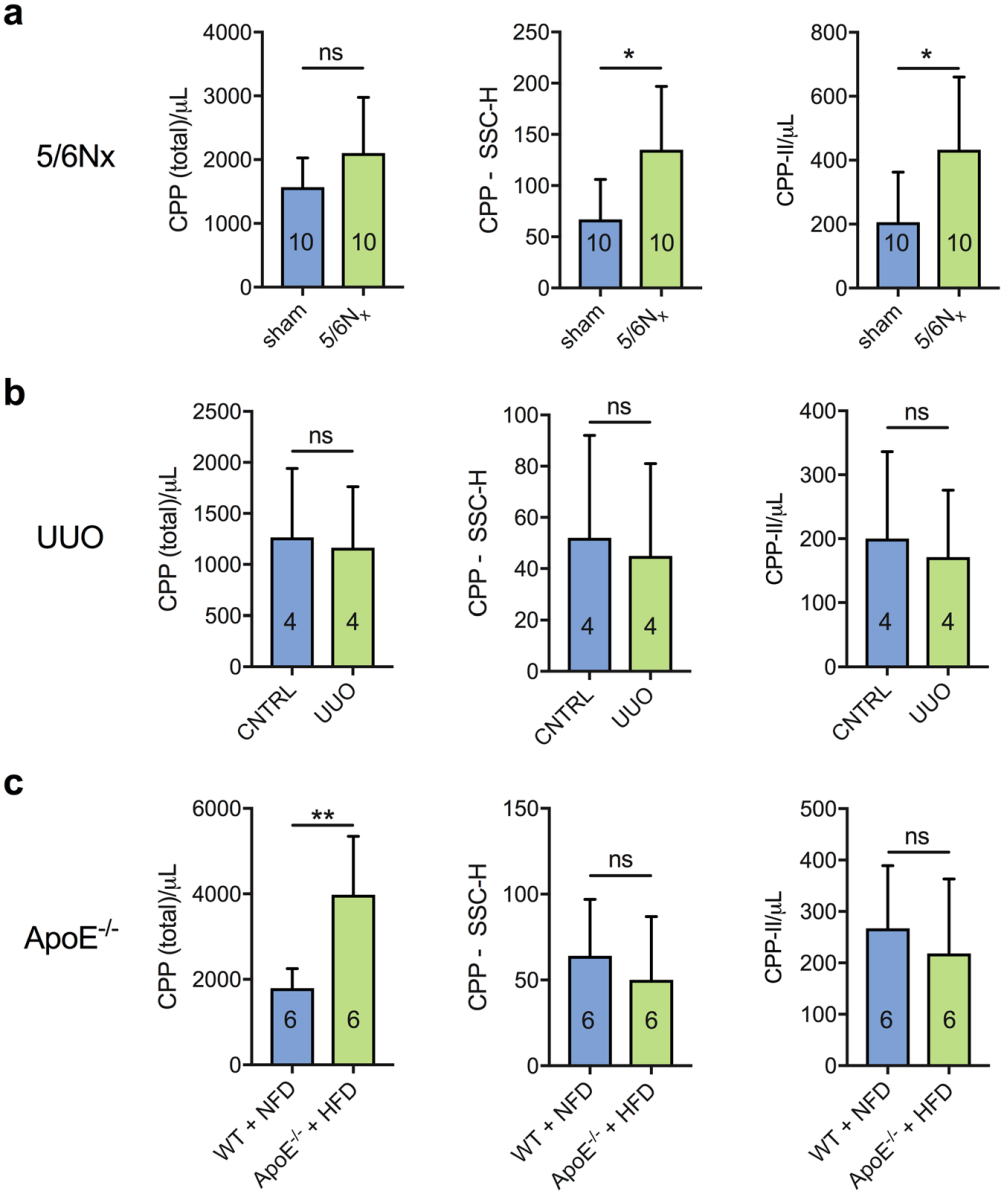
Quantitative flow cytometric analysis of serum calciprotein particles in uraemic and non-uraemic rodents. Total CPP, CPP SSC-H and CPP-II levels in (**a**) rats with subtotal nephrectomy, (**b**) mice with unilateral ureteric obstruction and, (**c**) high-fat fed ApoE-knockout mice, each compared to their respective control animals. P-values shown for pairwise comparisons made with unpaired Welch’s *t* test: ns, not significant (>0.05); *P < 0.05, **P < 0.01, *** < 0.001. Sample sizes (n) as indicated. ApoE, apolipoprotein E; CNTRL, control; CPP, calciprotein particles; HFD, high-fat diet; NX, nephrectomy; SSC, size scatter; UUO, unilateral ureteric obstruction.

To assess the broader utility of the method, we analysed archived serum samples obtained from several different animal models using the same analytical workflow (Fig. 5). As in adenine-treated rats, increased SSC-H and CPP-II levels were found in mice following subtotal nephrectomy (5/6Nx) compared to sham-operated controls (Fig. 5a). While total CPP concentrations were elevated in adenine-treated rats they were unchanged in 5/6Nx animals, suggesting increased ripening but not enhanced formation in this model. In mice with unilateral ureteric obstruction(UUO), on the other hand, flow cytometric parameters did not differ from unoperated littermates (Fig. 5b), consistent with the absence of systemic changes in mineral metabolism described in this model of experimental renal fibrosis^50^.

In an unrelated model, high-fat fed ApoE^−/−^ mice showed increased total CPP levels compared to wild-type animals fed standard chow (Fig. 5c). Since CPP-II levels and SSC-H were unchanged, this implies that the elevation in CPP was related to increased CPP-I.

## Discussion

In summary, we have developed a rapid homogeneous flow cytometric method for the detection and quantitation of individual mineral-containing nanoparticles in human and animal serum. To enhance sensitivity and specificity we have employed fluorescence-based thresholding as a signal trigger, which can reliably distinguish fluorescent particles from non-fluorescent background and free unbound dye without the need for a separation step. This method also allows the discrimination of membrane-bound from membrane-free particles and different mineral phases (amorphous CPP-I vs. crystalline CPP-II). Critically, the method has been optimised for use on a conventional benchtop instrument, without the need for manual hardware adjustments or dedicated microparticle flow cytometer. Therefore, we anticipate that the method will be readily accessible to others without the need for an experienced technician capable of complex modifications and calibration.

Using this method, we could demonstrate a consistency in findings across studies of CKD patients and in uraemic animal models, which showed that renal dysfunction is associated with enhanced ripening of CPP to the crystalline CPP-II state. In dialysis patients, circulating CPP levels were associated with markers of inflammation, bone metabolism and mineral metabolites, while in adenine-treated rats, chronic phosphate dietary loading appeared to exacerbate the changes in CPP seen with uraemia.

### Links between CPP, bone and lipid metabolism

Although the current flow cytometric assay for CPP described here employs an entirely different analytical approach, it is reassuring to observe that variables associated with CPP Fet-A levels determined using the ELISA-based method, namely, phosphate, bone turnover markers (C-terminal telopeptides) and inflammatory cytokines^8, 10^, remained significantly correlated with the readout from this next generation assay in a different cohort of CKD patients. However, the new method also revealed novel associations which were not apparent using the older method; levels were found to be higher in patients with type 2 diabetes and correlated strongly with serum calcium and FGF23 concentrations.

The finding of the association of CPP with FGF23 is particularly noteworthy, as the mechanism(s) driving elevations in FGF23 in CKD are poorly understood^51^. Bone-derived FGF23 has emerged as a possible ‘phosphate-responsive’ mediator of cardiovascular disease in CKD, but its secretion, while associated with dietary phosphate intake, paradoxically does not appear acutely or directly regulated by extracellular phosphate levels *in vitro* ^52^ or *in vivo* ^53^. Interestingly, recent *in vitro* studies have revealed that CPP stimulates the secretion of FGF23 in cultured osteocytes^54^. Thus, it is tempting to speculate that CPP may at least partly mediate the stimulatory effects of dietary phosphate loading on FGF23 via increased particle formation or ripening. Further work is needed to explore this attractive hypothesis directly. Corroborating these findings, CPP Fet-A levels have recently been correlated with intact FGF23 in a diabetic cohort (with preserved renal function) and may therefore have significance beyond ureamia as described here^26^.

Matsui *et al*. reported that a high molecular weight fetuin-A-containing mineral fraction was observed in the serum of adenine-treated rats but not in animals fed a control diet^11^. Interestingly, CPP became undetectable in uraemic animals given alendronate. This is consistent with the earlier work of Price *et al*., who showed that administration of various bone-modulating agents had profound effects on circulating levels^47, 55^, implicating the involvement of the skeleton as a source and/or sink for CPP. While the association of CPP levels with multiple bone-related cytokines (including FGF23) noted here is certainly suggestive of strong links to bone metabolism, further work is needed to evaluate the direct contribution of bone turnover to CPP formation in uraemia, and conversely, to consider the effects of circulating mineral nanoparticles on the bone itself. The purely associative nature of these findings, and the lack of adjustment for potential confounders due to the small sample size, precludes us from making more definitive comments about the interaction of CPP with bone.

Another plausible explanation for the apparent link between CPP and bone is that the interaction might be indirect, and mediated by other common factors, such as changes in inflammatory signaling^56, 57^. However, counter to this argument, the strength of the association of total CPP concentrations with both FGF23 and sclerostin was only very modestly attenuated by adjustment for IL-1β (r_p_ = 0.33; r_p_ = 0.25, respectively) or IL-6 (r_p_ = 0.35; r_p_ = 0.22, respectively).

While our studies in adenine-treated rats and in 5/6 nephrectomised mice indicated a common pattern of changes in CPP ripening following induction of uraemia, substantial elevations in total CPP levels were additionally observed in the adenine model. Since the mineral and protein content of the chow fed to these animals was identical we can exclude differences in diet as a potential cause. Other possibilities included increased production (e.g. from bone) and/or impaired clearance. Clearance-related effects would appear less likely, as in normal mice, CPP clearance is mediated by Kupffer cells of the liver and marginal zone macrophages in the spleen, without involvement of the kidney^58^. While it might be anticipated that changes in bone turnover could account for the elevations in CPP-II, as suggested by our studies in human CKD, it is notable that these two uraemic animal models have quite distinct bone histomorphometry. In adenine-treated rats high-turnover bone disease occurs, but in contrast in 5/6Nx animals there are only relatively subtle changes in the mineralisation surfaces and trabecular number, without accompanying disturbances in turnover^59^. The increased levels of CPP-I seen in adenine-treated rats may therefore provide a surrogate for the increased bone turnover observed in this model. Clearly studies comparing the clearance and tissue disposition of CPP in animals with intact renal function to those with uraemia are needed to clarify this issue, in addition to longitudinal studies tracking changes in bone turnover with circulating CPP.

The finding of increased CPP-I in high-fat fed ApoE^−/−^ mice is also noteworthy. Although this finding may partly reflect differences in feed: high-fat chow is casein-based with phosphate in a more bioavailable form than grain-based control chow; this is unlikely to fully explain this observation as CPP levels were significantly higher in ApoE^−/−^ mice than in high-phosphate fed control rodents. Urine samples were not available to assess fractional phosphate excretion as a proxy for phosphate loading in our study, so we were unable to exclude relative phosphate loading as a contributor to the augmented CPP levels observed. As an alternative explanation, we speculate that reduced clearance of CPP may also contribute to the elevated levels in these animals, due to the competing effects of accumulating ligands, like modified LDL particles, which share the same scavenger pathways. Indeed, the same receptor (class A scavenger receptor) is involved in the endocytosis of CPP and pro-atherogenic oxidised LDL and acetylated LDL^58, 60^, which are known to accumulate in high-fat fed ApoE^−/−^/mice^61^, and have been shown to compete for binding *in vitro* ^58^. Oxidised LDL levels are also found to be elevated in patients with CKD, despite typically normal (unmodified) LDL cholesterol levels^8^. Whether this holds significance for atherogenic processes remains to be seen but it is interesting to note that Herrmann *et al*. showed that CPP are taken up avidly by lesional macrophage in the carotid arteries of ApoE^−/−^/Fetuin-A^−/−^ mice^58^.

### Intestinal origin of circulating CPP and responsiveness to dietary mineral loading

The *in vivo* data presented here provide the first direct evidence that circulating CPP are responsive to changes in dietary mineral and highlight the disparate effects of loading on CPP ripening in animals with renal dysfunction compared to those with preserved renal function. That CPP increase with dietary phosphate content is entirely consistent with recent diurnal studies of circulating CPP in human diabetic subjects with normal renal function, where the authors noted a transient rise in levels after meals^26^. Importantly, in high-phosphate fed non-uraemic controls, we found that the rise in serum CPP-I was not accompanied by any detectable change in serum calcium and phosphate concentrations. This implies that quantitation of CPP levels may provide a better readout of dietary mineral exposure than these conventional measures.

More fundamentally this data also speaks to the likely source of at least a proportion of the circulating mineral nanoparticle pool. Indeed, if serum calcium and phosphate levels are maintained within normal limits, how can CPP formation be appreciably altered in the post-prandial state? Elegant studies by Powell and colleagues^36^ may provide one plausible explanation. They delineated an endogenous intestinal nanomineral transport system in which abundant ~100 nm amorphous calcium phosphate particles, similar in appearance to CPP-I, are formed spontaneously in the distal small bowel from the high concentrations of calcium (~4 mM) and phosphate (>10 mM) present in intestinal secretions^62^. These particles bind various macromolecules (proteins and peptidoglycans) present in the gut lumen, and are subsequently transcytosed across the epithelial barrier, principally via M cells of Payer’s patches, to antigen-presenting immune cells resident in the intestinal tissue. This process appears to occur constitutively, even in the fasting state, generating ~2 × 10^14^ particles/day, and putatively has a role in generating immunological tolerance^36^. Indeed, it has been known for decades that both endogenous and synthetic particles (<250 nm diameter) can efficiently translocate across the small gut epithelium via small intestinal lymphoid aggregates and, to a lesser extent, villous tissue^63–65^. We speculate that dietary loading with mineral may overwhelm this pathway resulting in spillover of particles into the circulation via the portal system.

Intriguingly, Thomas and colleagues^53^ recently reported that acute oral phosphate by gavage induced elevations in PTH and sustained phosphaturia while observing no change in serum calcium, phosphate or FGF23, thus the trigger of this adaptive response was uncertain. In this regard, it is worth noting that the experimental conditions of the original *in vitro* studies showing the stimulation of PTH secretion in parathyroid glands by phosphate, independent of ionised calcium, would be expected to generate CPP *in situ* ^66^. Therefore, studies investigating the role of CPP in triggering PTH secretion seem warranted. Indeed, as shown here in dialysis patients, CPP levels are strongly correlated with PTH in CKD^9^. Thomas *et al*. also made another highly relevant observation relating to the disposition of phosphate loads administered via either oral or intravenous routes^53^. In their study, urinary excretion at 4 h post gavage only accounted for approximately 50% of the administered load, at which point serum phosphate was unchanged (with oral loading) or normalised (with an intravenous load), and tissue phosphate levels were similar to vehicle-treated animals^53^. Since other routes of elimination (e.g. gastrointestinal) do not appear to quantitatively contribute in this situation^67^, these data are compatible with the existence of an extracellular compartment that can buffer such loads. Indeed, this is entirely consistent with much earlier intravenous loading studies by Gersh^68^, who reported the appearance of a colloidal mineral phase in the serum of dogs to which he had administered supraphysiological amounts of calcium and phosphate salts.

Overall, given the rapid clearance of CPP from the circulation^58^ and the concentrations of calcium and phosphate needed to drive CPP formation in extracellular fluid in a biologically relevant timeframe^4^, the gut represents the most likely origin of the post-prandial CPP surge and circulatory pool. From a teleological perspective, we postulate that expansion of the CPP-I pool may serve as a temporary buffer for the increased mineral flux, helping to maintain ionic homeostasis, until the particle mineral load can be safely metabolised and/or renally excreted. While this theoretical model implies a physiological role for CPP-I formation and clearance, our data also highlights that disturbances in mineral handling found in the uraemic state (e.g. hyperphosphataemia) favour nanocrystal ripening and the accumulation of potentially pathologic CPP-II.

Multiple studies in uraemic rodent models attest to the pathological effects of dietary phosphate loading on the heart, vasculature and kidneys. Although the mechanism(s) by which phosphate imparts these effects remains to be fully elucidated at the molecular level, phosphate overload has nonetheless been implicated as a major driver of vascular calcification, endothelial dysfunction, cardiac hypertrophy, tubulointerstital fibrosis and systemic inflammation in these uraemic animals [reviewed in refs 69–71]. Due to the biological plausibility of elevated phosphate levels directly contributing to disease, and strong associative evidence, interventions aimed at restricting phosphate intake or reducing intestinal absorption with binders have formed a mainstay of therapy for patients with advanced CKD^72^. However, while the control of dietary phosphate has proven effective in attenuating cardiovascular sequelae in animals^73–75^, and has been associated with a survival advantage in some studies of end-stage renal disease patients^76–79^, this effect appears largely independent of effects on serum phosphate concentration itself. Indeed, serum phosphate concentrations rise relatively late in the progression of CKD^80^, despite cardiovascular disease often being evident much earlier, and at best show very weak correlations with dietary phosphate intake if at all^81^. Nonetheless, that phosphate levels remain linked to events and outcome in CKD^82–84^, albeit only modestly after adjustment for confounding^85^, suggests that factor(s) related to phosphate may still have pathophysiological importance as well as prognostic significance. Since substantial data from both *in vitro* and *in vivo* studies already suggests that calcium phosphate nanocrystals, rather than phosphate itself, may mediate the deleterious effects of phosphate loading on the body^13–15, 86^, we speculate that an inability to clear CPP derived from dietary mineral intake, as appears to be the case in CKD, may result in ripening to the crystalline state and consequently drive some of the pathological processes previously attributed to ‘phosphate’. Clearly, further work is needed to confirm this hypothesis before we can consider CPP as a true phosphotoxin. Moreover, it will be important to understand how uraemia might affect the absorption and clearance of CPP and whether these pathways are amenable to intervention. From a practical point of view, a further corollary of these findings is that future clinical studies will need to carefully consider the prandial status of participants.

## Conclusion

Future studies are needed to evaluate the effect of dietary modification on CPP in human CKD and to assess more directly the relevance of intestinal and bone-derived CPP as a source of these circulating particles *in vivo*. Prospective analyses evaluating the association of flow cytometric-derived CPP measurements with vascular measures (e.g. calcification) and hard endpoints, as demonstrated using the ELISA method, are also needed to further assess the prognostic performance ﻿of this assay.

Overall, the new method described here represents an important step forward in analytical capability over the existing state-of-the-art ELISA-based techniques, allowing specific, fully quantitative and discriminative analyses of mineral-containing nanoparticles in fluids from humans and animals. From a clinical perspective, CPP testing by flow cytometric analysis may enhance our understanding of mineral handling in CKD and provide a novel indicator of therapeutic efficacy for interventions targeting the CKD-mineral bone disorder.

## Methods

### Human studies

Participants were enrolled (January 2014 to September 2016) in an observational study of Fetuin-A Levels in Systemic disease and Kidney Impairment (FLEKSI) conducted at the Royal Melbourne Hospital. These included 40 prevalent haemodialysis patients (“HD” group) and 40 patients undergoing peritoneal dialysis (“PD” group). All dialysis patients were stable, without evidence of inter-current illness, and were achieving small molecule clearance targets. 8 of 40 PD patients had residual renal function. PD patients were free from peritonitis for at least 6 months. All HD patients were anuric and receiving conventional maintenance HD thrice weekly (on average 4 hours per session) with a standard dialysis solution containing 1.25 mmol/L calcium, 0.5 mmol/L magnesium and 35 mmol/L bicarbonate and using a Gambro Polyflux 210 diaylser (Polyamix membrane). Dialysate was regularly monitored for impurities (<0.1 CFU/mL, <0.03 EU/mL endotoxins). PD fluids used contained 1.25 mmol/L calcium. Exclusion criteria included known pregnancy, age less than 16 years or greater than 90 years. A detailed drug history was recorded on all patients, including over the counter preparations. Medication use in these patients at the time of sampling is summarised in Supplementary Table S1. 40 healthy adult subjects were enrolled from staff and volunteers. Control subjects had no history of cardiovascular disease (exclusion criteria: previous myocardial infarction, stroke, heart failure, or receiving lipid-lowering/antihypertensive therapy), type 2 diabetes mellitus, malignancy, recent infection or trauma, or with known renal disease. Prandial state was uncontrolled and the timing of sampling was not standardised to facilitate recruitment of patients from AM and PM haemodialysis sessions, thus dietary effects cannot be assessed.

Venous bloods were taken for standard clinical care (serum calcium, phosphate, magnesium, bicarbonate, albumin, plasma PTH, 25-hydroxy-vitamin D) and for the assessment of mineral-containing nanoparticles (CPP, MBP), inflammatory and bone markers. In HD patients, samples were taken prior to starting dialysis and circuit anticoagulation. Blood was collected into 6 mL plain (#367837) and K_2_-EDTA (#367863) tubes (both BD Biosciences, San Jose, CA, USA) using standard phlebotomy techniques. Blood samples were allowed to stand for 60 minutes and then centrifuged at 3000 *g* for 15 min at room temperature (Eppendorf 5430R, A-4-38 rotor or 5415 D, F45-24-11 rotor) to pellet cells. Aliquots were then stored at −80 °C until batched analysis or processed immediately for nanoparticle analysis as detailed below. All analyses were performed on samples that had not been thawed previously. Standard biochemical analysis was performed using a routine automated analyser. Serum inflammatory markers (IL-1β, IL-6 and TNF-α) were measured in technical duplicates using the human inflammatory cytokine bead array (CBA) kit (#551811), according to the manufacturer’s instructions. Data were analysed using FCAP Array Software Version 3.0 (BD). Serum bone markers (DKK-1, osteocalcin, osteoprotegerin, osteopontin, sclerostin) were measured in technical duplicates using the MILLIPLEX luminex xMAP human bone magnetic bead panel (#HBNMAG-51K; EMD Millipore, Bayswater, Australia) according to the manufacturer’s instructions. Serum CPP Fet-A reduction ratios were determined as described previously^7^. Patients gave written informed consent, and the study was approved by local regional ethics committee (Melbourne Health Research and Ethics Committee ref: 2012/141) and was conducted in accordance with the Declaration of Helsinki.

### Animal studies

To assess the performance of the method in animals, we performed *de novo* studies in rats using the adenine-induced renal failure (AIRF) model as previously described^49^. Sixty male Wistar rats (8-week old; 250–325 g) were obtained from the local breeding colony. Animals were housed individually in a temperature- and humidity-controlled environment (25 °C at 25% humidity), with a 12/12 h light/dark cycle and had unlimited access to water and standard chow (Harlan-Tekland 22/5 diet 8640: 1.13% Ca, 0.94% P, 3.0 IU/g cholecalciferol, 22.6% protein). After 1 week of acclimatisation, the rats were randomly allocated into 2 groups (30/group). Uraemia was induced in one group of animals with an adenine-supplemented diet: 0.75% for 2 weeks, then 0.5% for a further 2 weeks. We employed this tapered dose to avoid severe renal failure and mortality. The remaining animals received standard chow throughout this 4 week period and served as the normal renal function Control group. Uraemic and Control groups were then further divided into 2 subgroups (15/group) and were allocated to receive either standard chow (as above; normal phosphate diet, NPD) or a high phosphate-containing feed (0.6% Ca, 1.21% P, 18.6% protein from grain, 2.2 IU/g cholecalciferol; high phosphate diet, HPD) for a further 6 weeks. AIRF groups were balanced based on body weight (monitored weekly), serum creatinine and phosphate. Control animals were allocated according to body weight. No animals died during the study. At the beginning of week 0 (i.e. prior to the commencement of dietary adenine), and end of weeks 4 and 10, animals were placed in metabolic cages for the collection of urine. Morning blood samples were also taken at the same timepoints. Blood samples were allowed to clot for 60 min at room temperature and then centrifuged (3,000 *g*, 10 min) to separate serum from cells. Urine samples were collected on wet ice for 8–14 (max) hours and adjusted to estimate 24 hour clearance. Urinary sediment was removed by centrifugation (5,000 *g*, 15 min, 4 °C) prior to storage. Animals were not offered food during the urine collection period. Serum and urine aliquots were stored at −80 °C until analysis or processed further for nanoparticle analysis (as described elsewhere). At the end of week 10, rats were anesthetised and sacrificed by exsanguination via aortic puncture.

Calcium, creatinine and urea were measured using colorimetric kits from BioAssay Systems (DICA-500, DICT-500 and DIUR-500, respectively). Phosphate was determined using a BioVision Systems colorimetric kit (K-410). Creatinine clearance (CrCl) was estimated according to the formula: urine creatinine concentration × 24 hour urine volume/serum creatinine concentration. The fractional excretion of phosphate (FeP) was calculated according to the formula: urine phosphate concentration × serum creatinine concentration/serum phosphate concentration × urine creatinine concentration ×100%. Intact FGF23 and PTH were measured by ELISA according to the manufacturer’s instructions (Immutopics Inc, San Clemente, USA). Serum IL-1β, IL-6 and TNF-α concentrations were measured by ELISA (R&D Systems, Abingdon, UK).

Additionally, we accessed archived serum samples from experiments encompassing both uraemic and non-uraemic pathologies, together with their respective controls, from which blood samples were collected at planned sacrifice. Experimental designs followed previously published reports^87–90^. In rats with subtotal nephrectomy (5/6Nx), renal insufficiency was induced by a two-step surgical procedure involving uninephrectomy followed by ligation of 2/3 branches of the renal artery supplying the remnant kidney. Control animals underwent sham surgery. Blood was taken at 6 weeks post-surgery. Animals were fed standard chow throughout as above. Unilateral ureteric obstruction (UUO) was performed in male C57BL/6J mice (8-10-week-old). Blood was taken 3-days post-surgery. A parallel control group consisted of un-operated male animals. UUO and control mice received standard chow throughout. A final group of animals consisted of male ApoE^−/−^ mice (C57BL/6J background) that were fed a Western-style high-fat diet (21% fat, 0.21% cholesterol; #RD12079B) from 5 to 19 weeks of age. Same aged male C57BL/6J mice given standard chow were used as controls. All serum samples had been stored at −80°C and were analyzed without previous freeze/thaw cycles.

All experimental protocols were approved by the Monash University Animal Ethics Committee, and were carried out in accordance with the *Australian Code of Practice for the Care and Use of Laboratory Animals for Scientific Purposes*.

### Sample preparation and particle labelling for flow cytometric analysis

As described previously^27^, additional processing steps are required to prepare serum samples for mineral nanoparticle analysis. Briefly, 50 μL serum or plasma sample were diluted 1:5 in twice-0.22 μm filtered (Millex-GP PES membrane; #SLGP033RS; Merck Millipore, Cork, Ireland) Tris-buffered saline (TBS, pH 7.4) and centrifuged again to pellet large debris (10,000 *g*, 15 min, 4 °C; Eppendorf 5430R, FA-45-24-11-HS rotor). These steps preserve the crystallisation state of the particle and reduce background contaminants. Supernatants were then collected and stored at −80 °C until batched staining and analysis. OsteoSense 680EX fluorescent dye (#NEV10020EX; Perkin Elmer, Boston, MA, USA) was reconstituted in 1 mL twice-0.22 μm filtered TBS (stock concentration: 24 μM), re-filtered, and centrifuged at 30,000 *g* for 1 h at 4 °C to remove any dye aggregates. PKH67 green fluorescent cell linker midi kit for general cell membrane labelling was obtained from Sigma (#MIDI67-1KT; St. Louis, MO, USA). For staining of mineral-containing nanoparticles, 200 μL supernatants were mixed with 250 μL OsteoSense 680EX reagent (1:50 diluted stock in TBS) in a microfuge tube (safe-lock, Eppendorf) for 50 min at 4 °C protected from light and with gentle rotational mixing (MACSmix tube rotator, #SN 4747, Miltenyi Biotech, Bergisch Gladbach, Germany). This incubation mixture was then transferred to another microfuge tube containing 25 μL PKH67 reagent (PKH67 dye diluted 1:100 in diluent C; 0.22 μm double-filtered; prepared immediately prior to staining), vortexed, and incubated for a further 10 min at 4 °C, protected from light, before sample acquisition. This volume provides sufficient sample for measurements in triplicate at a flow rate of 50 μL/min.

### Preparation of CPP standards

CPP-I and CPP-II were prepared from pooled human serum as described before^45^. Briefly, serum was pre-cleared of nanoparticulates using a stepped centrifugation protocol: (1). 10,000 *g*, (30 min, 4 °C) to sediment large particles, (2). 100,000 g (2 h, 4 °C; Optima L-100 XP, CF32 Ti rotor set) to pellet all other particulates. Per 10 mL CPP mixture, the following solutions were added in order: (1) 1 mL 140 mM NaCl solution; (2) 4 mL pre-cleared serum; (3) 2.5 mL phosphate solution: 19.44 mM Na_2_HPO_4_, 4.56 mM NaH_2_PO_4_, 140 mM NaCl, 25 mM Tris (pH-adjusted with 10 M NaOH to 7.40 at 37 °C); (4) 2.5 mL calcium solution: 40 mM CaCl_2_, 140 mM NaCl, 25 mM Tris (pH-adjusted with 10 M NaOH to 7.40 at 37 °C) and incubated at 37 °C with gentle mixing. CPP-I and CPP-II were isolated from the mix at 1 h and 12 h, respectively, using high-speed centrifugation (30,000 *g* for 2 h at 4 °C). Pellets were then washed three times in ice-cold TBS and collected by centrifugation. CPP were then resuspended in 500 μL pre-warmed TBS and used fresh. To generate OsteoSense680EX -labelled CPP, 25 μL of the resuspended pellet was added to 75 μL stock OsteoSense 680EX reagent with mixing for 2 h at 4 °C in the dark, before isolating particles by centrifugation as above. Actual particle concentrations were determined by NTA as described below. All chemicals were analytical grade and purchased from Sigma. All solutions were double 0.22 μm-filtered before use.

### Flow cytometry of mineral-containing nanoparticles

Background information regarding the use of light scattering and fluorescence parameters in the study of nanoparticles and the quantitation of individual mineral-containing nanoparticles can be found in the Supplementary information.

Samples were run on a BD FACSVerse flow cytometer. This is a 3 laser (405, 488, 640 nm) benchtop instrument configured with vacuum-driven, high-sensitivity fluidics, a cuvette-based flow cell (gel-coupled by refractive index matching to the objective lens), high-performance PMTs and detection (emission) optics, and a flow sensor option for accurate volumetric measurements. For excitation optics, the laser beam is elliptically focused to a cross-sectional area 9 × 63 μm^2^. On this instrument, SSC resolution enables separation of >200 nm polystyrene beads from noise, while 110 nm fluorescent polystyrene beads can be resolved from noise using fluorescence triggering. 0.22 µm-filtered MilliQ water was used as sheath fluid. The instrument was operated using BD FACSuite software (version 1.0.5). Raw FCS files were acquired and imported into FlowJo LLC version 10.1 revision 3 (Ashland, Oregon, USA) for analysis.

After laser warmup, extensive washing must be undertaken to reduce the high background. We used a program of sequential 10 min runs with (1) MilliQ water; (2) FACSClean (BD, #340345); (3) MilliQ water; (4) FACSRinse (BD, #340346); (5) MilliQ water, all at high flow rate (120 µL/min), then switching to high-sensitivity mode fluidics mode and running water for a further 20 min during which time detectors stabilise and events fall to <80/s, with an applied SSC voltage of 486 V and thresholding at 200 V.

On our system, APC-Cy7 was the closest spectral match to the trigger signal fluorescence of OsteoSense 680EX. Therefore, events were triggered on the red 640 nm laser (44 mW) using the APC-Cy7 detector (783/56 filter). PKH67 fluorescence was detected using the FITC channel (527/32 filter). Performance of the instrument was verified on each day of use. We utilised two sets of nano-sized calibration beads (ApogeeMix: #1493; Spherotech Nano polystyrene size standard kit: #NPPS-4K) encompassing plastic spheres of different sizes and refractive indices to assess the sensitivity and resolving power of the light scattering optics. ApogeeMix beads were used to set the PMT voltages for light scattering. Bead characteristics and detection efficiencies are summarised in Supplementary Table S2. SPHERO Ultra Rainbow Calibration Particle Kit (#URCP-38-2K) was used to assess efficiency (Q) and optical background (B) parameters for the APC-Cy7 detector as described by Hoffman and Wood^91^. Using this protocol we derived the following parameters: Q = 0.49 per MESF, B = 37 per MESF, electronic noise RSD = 15.1, linearity 43 to 228881. SPEHRO Ultra Rainbow Fluorescent Nanospheres (100–300 nm; #URFP-02-2) were used to confirm fluorescence detection on the nanoscale. OsteoSense-labelled CPP-I/II controls were used to set PMT voltages for the APC-Cy7 fluorescence channel. Thresholds were set using reagent blanks (buffer/reagent no sample) as described in the text. Sample blanks (no reagents) were used to assess background fluorescence. These calibrations and controls were performed on each day of use and periodically throughout the measurement run. After calibration and threshold checks we ran the following cleaning program in high-sensitivity fluidics mode: 5 min MilliQ water, 5 min FACSClean, 5 min MilliQ water. With the fluorescence trigger threshold applied, reagent blanks were <20 events/min. Samples were previewed for 30 s before acquisition to allow stabilisation of the signal. Acquisition settings were held constant for all samples (60 s or ~30,000 events). Electronic abort rates were monitored and runs repeated at dilution if abort rates >10/s or if total event rates >500/s. All measurements were displayed in logarithmic scale and signal stability was assessed in real-time using SSC-H vs. time plots. To minimize sample carryover between samples, 5 sample injection tube (SIT) washes were performed following each sample acquisition. All samples were run in triplicate.

Preliminary sample stability studies using HD patient bloods (n = 4), indicated that total CPP counts were stable in serum for >4 h, but showed increased SSC-H after 24 h at room temperature (see Supplementary Fig. S9). As expected, CPP were unstable in EDTA-anticoagulated samples which demonstrated a significant decay in signal within 24 h.

Using HD serum samples, within-run coefficients of variation (CV) for CPP(total)/µL, CPP-II/µL and SSC-H were <5%, <8% and <10%, respectively, while between-run CV were <15% for CPP(total)/µL and CPP-II/µL.

For comparison, samples were also run on an Apogee A50 Micro Flow Cytometer (Apogee Flow Systems, Hertfordshire, UK) equipped with 50 mW 405, 488 and 638 nm lasers. Calibration was performed as for the FACSVerse. We used a sheath (MilliQ water) pressure of 150 mBar and 5 flush cycles. Measurements were recorded in log mode and noise levels were <0.5. Washing was performed using 10% bleach solution at a flow rate of 15 µL/min for 120 s, followed by filtered TBS with the same settings. Samples were run in duplicate at 1.5 µL/min (130 µL total) for 120 s or until the data buffer was full (5 × 10^6^ events), thresholding on APC-Cy7 fluorescence as described above.

### Nanoparticle tracking analysis

Nanoparticle tracking analysis (NTA) was performed on a Nanosight NS500 (Nanosight, Amesbury, UK) equipped with blue (488 nm) laser, sCMOS camera, and using NTA 3.2 analytical software. Samples were analysed at two dilutions (1 in 100; 1 in 1,000 in twice-0.22 μm filtered TBS) to encompass the linear working range on the instrument (10^6^ to 10^9^ particles/mL), each for 5 × 60 s captures, and the results averaged. Capture settings (camera level 11; slide shutter 890; slider gain: 146; frames/s:25; syringe pump speed: 50 μL/min) and detection settings (threshold: 4; blur: auto; max jump distance: auto) were kept constant for all analyses.

### Electron microscopy

Cryogenic-electron microscopy was performed as previously described^44^. Briefly, particles were pelleted from diluted serum by centrifugation (30,000 to 50,000 *g*, 2 h, 4 °C; as indicated in the text), washed three times in TBS (pH 7.4) and re-suspended in 0.22 μm filtered MilliQ water. Samples were plunged frozen in liquid ethane before observation on a Tecnai F30 (FEI, Netherlands) operating at 300 kV.

### Statistics

Variables were expressed as mean (SD) or median (25th–75th percentile) or as otherwise indicated. D’Agostino & Pearson’s omnibus test was used to assess normality. Non-parametrically distributed variables were natural log-transformed before further analysis. Comparison of cohort subgroups was performed using one-way ANOVA with Bonferroni correction or Kruskal-Wallis test with Dunns post-test for multiple comparisons as appropriate. Pearson’s correlation coefficients (r) were calculated to assess the association of flow cytometric parameters and other variables. Partial correlation coefficients (r_p_) were determined to control for mediating factors. Unpaired Welch’s t tests were used for 2 group comparisons. Deming regression and Bland-Altman plots were used to evaluate the agreement between methods. For the adenine rat study, linear mixed models were used to assess the change in measured variable from baseline. Group and time were included as fixed effects and a random intercept for each animal was included as random effects. To evaluate whether the change in measured variable from baseline differed by group, an interaction term for each group and time was introduced. Residual plots were inspected to look for deviations from normality and confirm model assumptions. Two-tailed P values of <0.05 were considered significant. Analyses were performed using Stata version 12 (StataCorp, College Station, Texas, USA).

## Electronic supplementary material

Supplementary information
