# Supplementary material for: A novel fluorescent probe-based flow cytometric assay for mineral-containing nanoparticles in serum
Source: Sci Rep. 2017 Jul 18;7:5686. doi: 10.1038/s41598-017-05474-y (PMC5515983; doi:10.1038/s41598-017-05474-y)
Supplement: Supplementary file 1 — Supplementary information [file 41598_2017_5474_MOESM1_ESM.pdf]

Supplementary information for

A novel fluorescent probe-based flow cytometric assay for mineral-containing nanoparticles in serum

Edward R. Smith[1,2*], Tim D. Hewitson[1,2], Michael M.X. Cai[1,2], Parisa Aghagolzadeh[3], Matthias Bachtler[3], Andreas Pasch[3], Stephen G. Holt[1,2]

[1] Department of Nephrology, The Royal Melbourne Hospital, Melbourne, Victoria, Australia

[2] Department of Medicine - Royal Melbourne Hospital, University of Melbourne, Melbourne, Victoria, Australia.

[3] Department of Clinical Research, University of Bern, Switzerland

* Corresponding author

Email: edward.smith@mh.org.au

Supplementary methods

Light scattering and fluorescence-based detection of nanoparticles by flow cytometry

Conventionally, forward scatter (FSC) intensity is measured in line with the laser beam and is considered to provide a measure of cell size, while side scatter (SSC) intensity is measured perpendicular to the beam and yields information about cellular complexity or granularity. While cells mostly scatter light in the forward direction, nanoparticles scatter light over much larger angles due to diffraction[1,2]. For most conventional instruments, SSC detectors employ a wider collection angle than FSC detectors (~47-133° vs. ~0.5-7°, respectively), and thus provide better resolution on the nanoscale[3,4]. SSC detectors also generally use more sensitive photomultiplier tubes than the photodiodes typically used for FSC detectors, enhancing sensitivity yet further[4]. Indeed, SSC is optimised to detect the scatter from multiple submicron organelles within the cell interior[3], not dissimilar on scale to the detection of nanoparticulates. On our instrument, a BD FACSVerse flow cytometer, a mixture of 450, 220 and 130nm polystyrene beads could only be resolved from each other using SSC, and not FSC (see Supplementary Fig. S1A), consistent with the better resolving power and sensitivity of SSC. Using the instrument's default settings, detection efficiencies (ratio of measured and known concentrations) were 1.0, 0.9 and 0.1±0.1, respectively, demonstrating that the majority of 130nm beads remained undetected when applying the minimum (system imposed) SSC threshold.

Operation of the instrument in a lower pressure high-sensitivity fluidics (HSF) mode, where the sample flow rate (50 vs. 120 μL/min) and sheath core stream velocity (2.7 vs. 5.4 m/s) are reduced compared to normal, improves hydrodynamic focusing of the sample stream and prolongs particle interrogation in the laser beam, thus allowing better separation of small, dimly stained particles from background noise[3] (see Supplementary Fig. S1B).

Scatter readings scaled to pulse height (SSC-H) rather than pulse area (SSC-A) also improved signal resolution (see Supplementary Fig. S1C), as pulse area is more related to particle path length through the laser beam than light scattering properties *per se*[5]. Using these settings, detection efficiencies of 1.0, 1.0 and 0.6±0.1 were obtained for 450, 220 and 130nm beads, respectively.

Importantly, SSC is influenced by the shape and optical properties of the particle (refractive index, geometry, internal structure)[2,3], as well as size, and therefore only provides an approximation and relative sizing of particles of the same composition. We show this here using a second set of calibration beads containing a mixture of unlabelled silica beads (refractive index, η=1.43) and green-fluorescent latex particles (η=1.59) of different sizes. As shown in Supplementary Fig. 1D and 1E, SSC is related to size and optical properties of the material being interrogated as 500nm latex particles of higher refractive index scatter more light (~10 times more) than less refractive 580nm silica beads. This is advantageous for the current application as different mineral phases not only have variable refractive indices[6], but are also much higher than for biological vesicles[7] (e.g. hydroxyapatite: η~1.64; vesicles: η=1.36-1.40).

Most conventional flow cytometers have limited ability to resolve particles <300-500 nm using light scattering alone, due to the overlap with noise generated by the buffer (e.g. Raman scatter from water), optics and electronics[2,3]. Our system can resolve 220nm polystyrene beads from noise using SSC-H (see Supplementary Fig. S1F). Discrimination of smaller, less refractive nanoparticles from noise can therefore only be made using fluorescence-based thresholding to trigger signal detection (see Supplementary Fig. S1G).

Quantitation of individual mineral-containing nanoparticles from serum

The addition of a flow sensor to the cytometer enabled accurate volume measurements during acquisition and the determination of absolute particle counts per unit volume. To determine the accuracy of this analysis, we compared volumetric measurements by flow cytometry with nanoparticle tracking analysis (NTA) on the Nanosight. NTA showed a strong correlation with particle counts of synthetic CPP-I and CPP-II below 10^6/mL (corresponding to ~500 counts/s). Above this concentration there was a non-linear relationship between estimates, with particle counts being relatively underestimated by flow cytometry (see Supplementary Fig. S5 online) and with significant electronic aborts (>100 events/s). Below this concentration, particle numbers diluted out linearly, and SSC-H remained relatively constant with abort rates <5 event/s (see Supplementary Fig. S5 online). Nonetheless, flow cytometry showed a strong negative bias compared to NTA particle concentrations, suggesting that the former may underestimate absolute levels by up to 10-fold, although readouts remained proportionate (see Supplementary Fig. S5 online). Of note, NTA analyses also suffer from calibration issues and may over-estimate the number of particles depending on the detection settings applied. In particular, NTA-derived estimates of particle concentration have been shown to be subject to substantial error when highly heterogeneous pools of particles are analysed[8]. Analogous dilution studies using human serum samples (n=10) showed that endogenous CPP also diluted out linearly, while SSC-H was stable to dilution (see Supplementary Fig. S5 online). That sample counts remained linear to dilution is an important finding as this argues against the 'swarm detection' of multiple particles passing through the laser beam at the same time and counting as a single event signal[4]. This appears to be the case for extracellular vesicles, where counts are dominated by larger vesicles (>1000nm), but smaller particles also contribute as 'swarms', if collectively exceeding the detection

threshold. Hence, counts of extracellular vesicles are grossly underestimated relative to actual concentrations (~1000-fold)[4]. The improvement in sensitivity and detection efficiency observed by switching to lower flow rates (yielding a smaller effective beam volume) and high dilution of samples both serve to reduce the likelihood of swarm detection, and further argue against this phenomenon in the current application where individual (single particle) detection is achieved. Given the similar size distribution of CPP and small extracellular vesicles (i.e. exosomes), it is likely that these substantial disparities in detection relate to large differences in optical properties such as refractive index.

Deming regression of volumetric analysis performed on the FASCVerse and an alternative 'dedicated' flow cytometer (Apogee A-50), showed a near linear relationship, although the FACSVerse demonstrated a mean negative bias of 26% i.e. yielding numerically lower values (see Supplementary Fig. S6 online). Equivalent scatter characteristics (medium angle light scatter; MALS) also correlated with SSC-H, albeit with substantial scatter. Thus, with appropriate calibration, the method is transferable to other instruments provided they have adequate sensitivity and performance.

References

1 Steen, H. B. Flow cytometer for measurement of the light scattering of viral and other submicroscopic particles. Cytometry A 57, 94-99, doi:10.1002/cyto.a.10115 (2004).

2 Kerker, M. *et al.* Light scattering and fluorescence by small particles having internal structure. J Histochem Cytochem 27, 250-263 (1979).

3 Shapiro, H. M. in Practical Flow Cytometry 101-223 (John Wiley & Sons, Inc., 2003).

4 van der Pol, E., van Gemert, M. J., Sturk, A., Nieuwland, R. & van Leeuwen, T. G. Single vs. swarm detection of microparticles and exosomes by flow cytometry. J Thromb Haemost 10, 919-930, doi:10.1111/j.1538-7836.2012.04683.x (2012).

5 Bohren, C. F. & Huffman, D. R. in Absorption and Scattering of Light by Small Particles (Wiley-VCH Verlag GmbH, 1998).

6 Moreno, E. C., Gregory, T. M. & Brown, W. E. Preparation and solubility of hydroxyapatite. Journal of Research of the National Bureau of Standards 72A, 773-782 (1968).

7 Beuthan, J., Minet, O., Helfmann, J., Herrig, M. & Muller, G. The spatial variation of the refractive index in biological cells. Phys Med Biol 41, 369-382 (1996).

8 Gardiner, C., Ferreira, Y. J., Dragovic, R. A., Redman, C. W. & Sargent, I. L. Extracellular vesicle sizing and enumeration by nanoparticle tracking analysis. J Extracell Vesicles 2, doi:10.3402/jev.v2i0.19671 (2013).

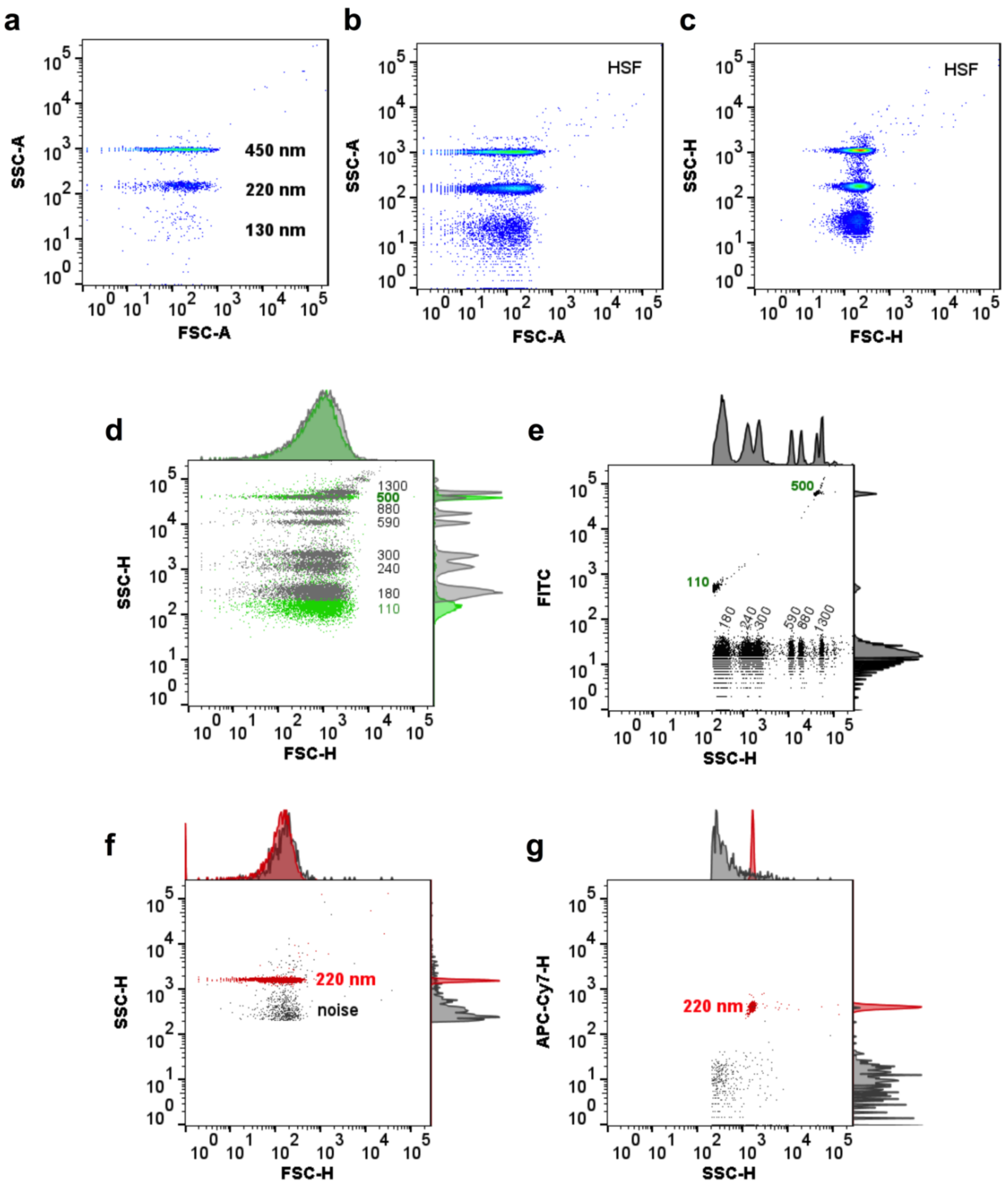

Fig S1. Performance of the BD FACSVerse flow cytometer for nanoparticle detection. (A-C) Dot plots showing the improvement in detection of 450, 220 and 130nm green fluorescent polystyrene nanospheres using SSC intensity with fluorescence-triggering, under (B) high-sensitivity fluidics mode and using (C) pulse height parameters, compared to (A) running in normal fluidics mode and pulse area readings. (D, E) Dot plots with adjunct histograms demonstrating the dependence of SSC on optical properties (e.g. refractive index) as well as particle size. The resolution of high-refractive green-fluorescent latex beads (110, 500nm; FITC) from less refractive non-fluorescent silica beads (180, 240, 340, 590, 880, 1300nm; grey) with SSC threshold (200V), using (D) light scattering, and (E) green (FITC) fluorescence. The separation of red fluorescent 220nm polystyrene beads from noise using by (F) SSC and (G) fluorescence with SSC threshold applied (200V). FSC, forward scatter; HSF, high-sensitivity fluidics; SSC, side scatter.

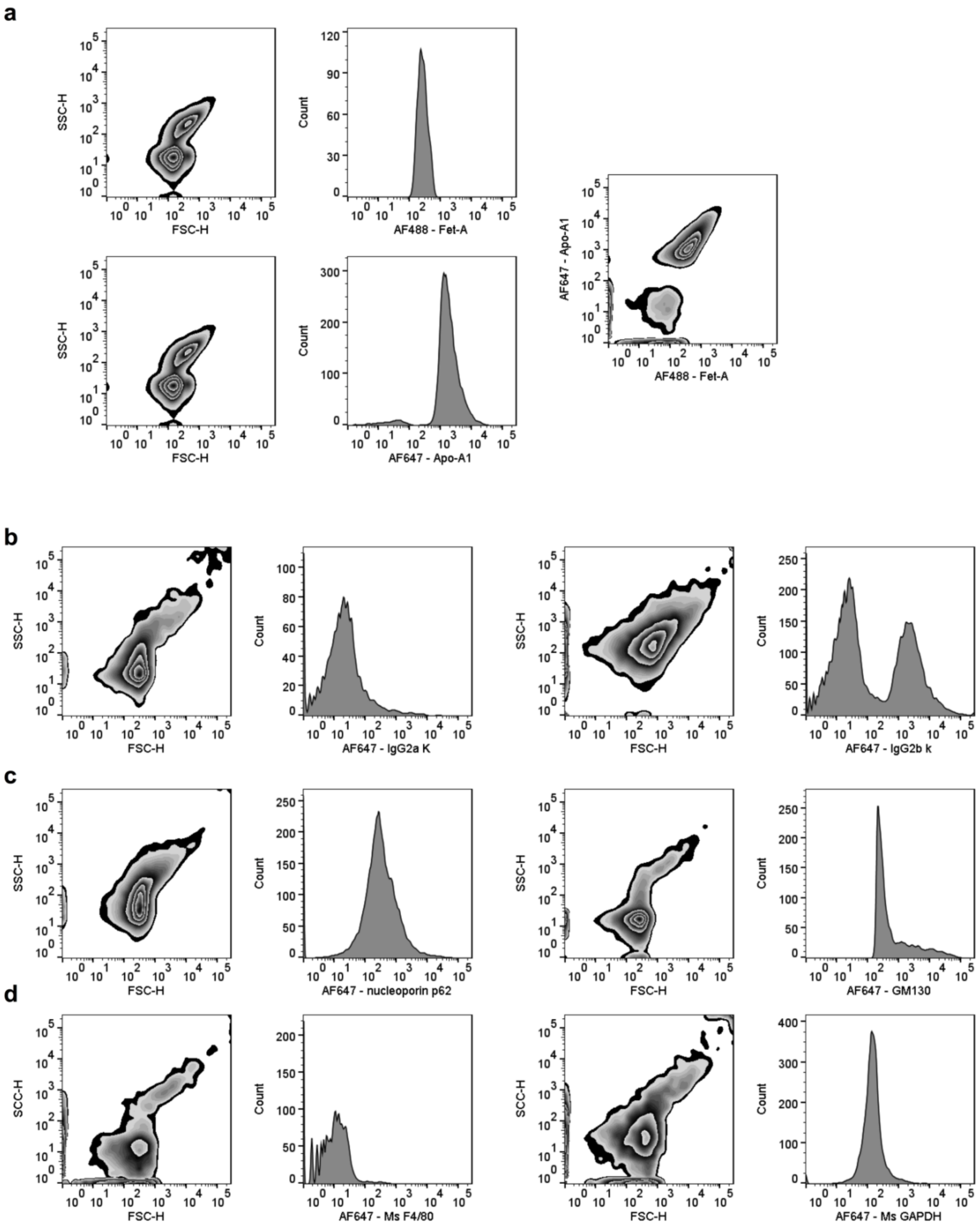

Fig S2. Antibody-mediated detection of CPP by flow cytometry. CPP were isolated from uraemic serum by high speed centrifugation (30,000 *g* for 1h at 4°C), and stained with fluorochrome labelled specific antibodies or control IgG (as indicated). Labelled particles were then separated from unbound antibody by centrifugation (as above) and measured by flow cytometry with fluorescence thresholding triggering on AF488. (A) Representative light scatter cytograms and fluorescence histograms of Fet-A-AF488 positive particles are depicted. Double-labelling of endogenous CPP with anti-Fet-A-AF488 and anti-Apo-A1-647 antibodies. Staining of Fet-A-AF488 positive particles with AF647-labelled (B) IgG_{2a} κ and IgG_{2b} κ antisera, (C) intracellular antigen-specific antibodies and (D) mouse-specific antibodies that do not cross-react with human samples. AF, Alexa Fluor; Apo-A1, apolipoprotein-A1; Fet-A, fetuin-A; FSC, forward scatter; SSC, side scatter

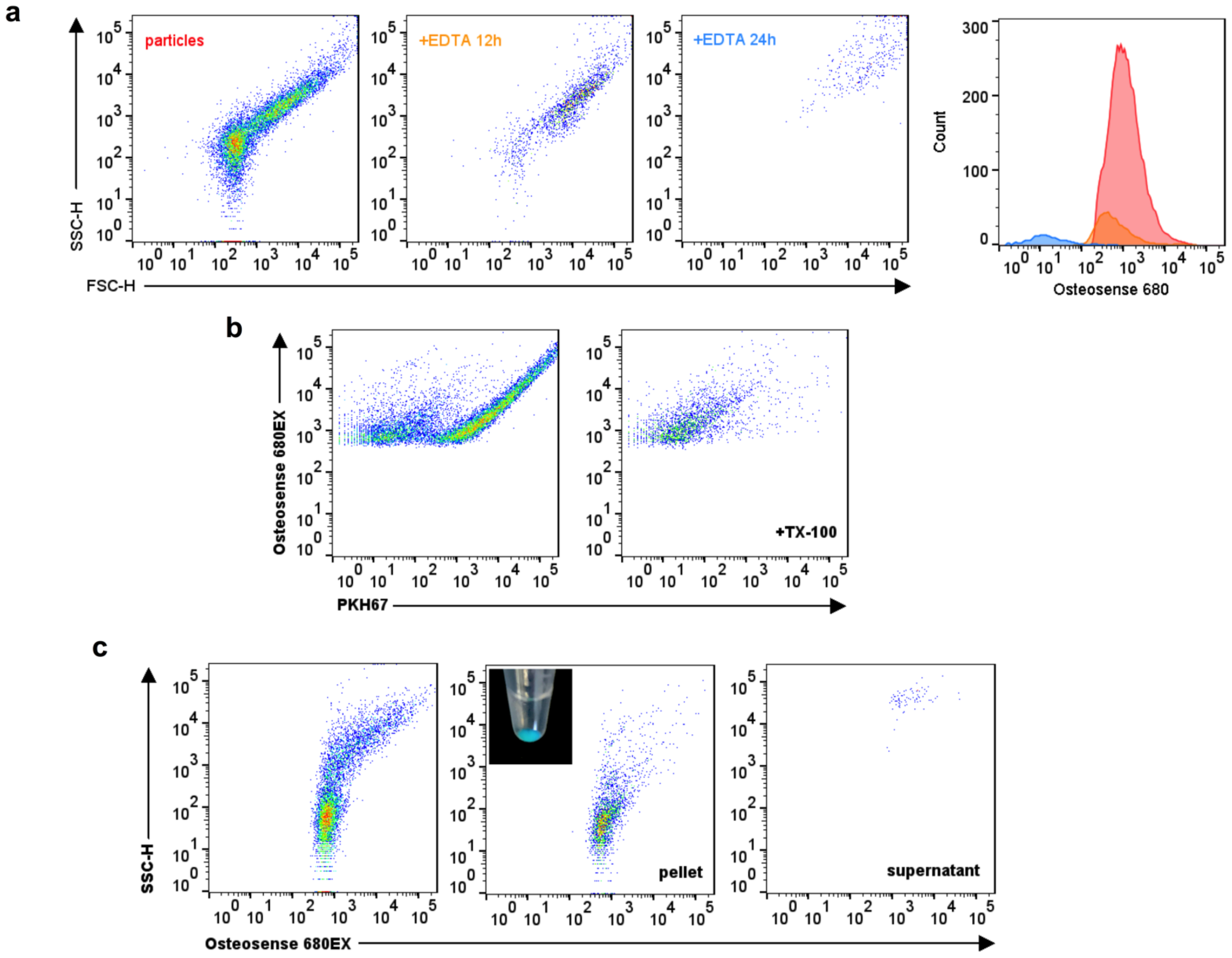

Fig. S3. Specificity of OsteoSense 680EX and PKH67 fluorescent probes. (A) Scatter dot plots of serum OsteoSense-positive particles and time-dependent dissolution in mineral chelator, EDTA. (B) Fluorescence dot plots showing dissolution of PKH67-positive serum mineral-containing nanoparticles with detergent (Triton-X100). (C) Dot plots showing the sedimentation of OsteoSense-positive particles with high-speed centrifugation. Inset depicts OsteoSense-stained pellet. EDTA, ethylenediaminetetraacetic acid; TX-100, Triton-X100.

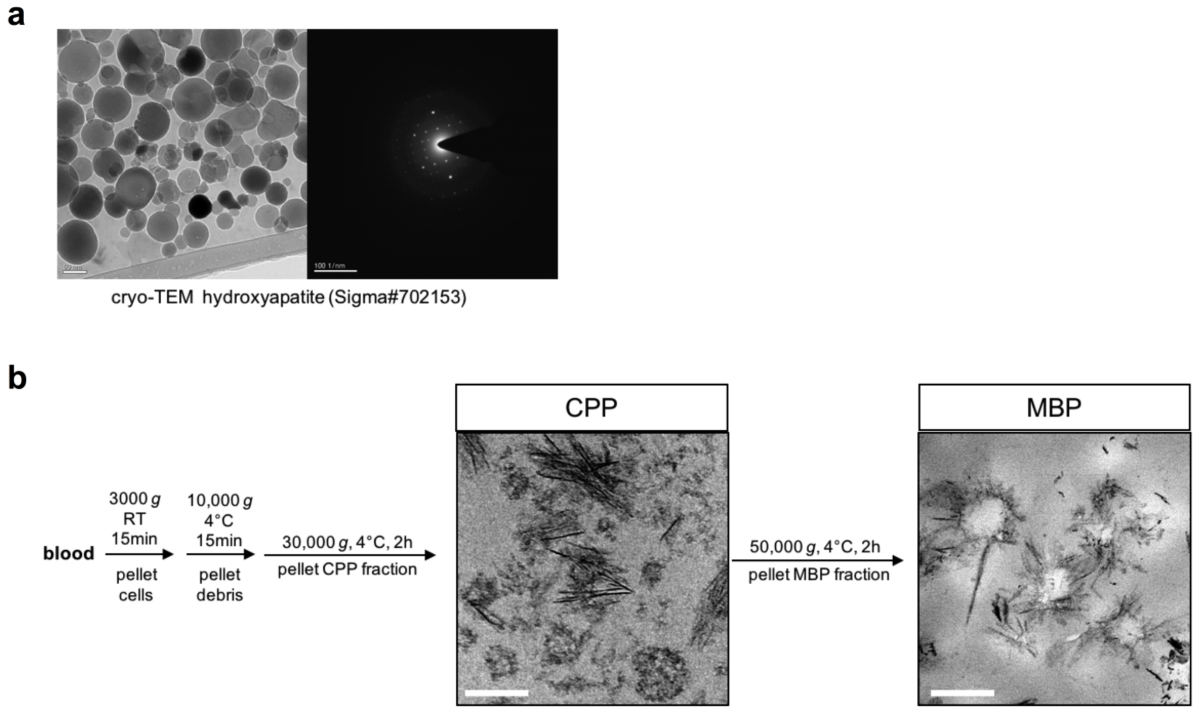

Fig S4. Microscopic analysis of mineral-containing nanoparticles. (A) cryogenic transmission electron micrograph and diffraction pattern of synthetic hydroxyapatite nanoparticles (<200nm) obtained from Sigma (#702153). Scale bar=50nm. (B) Schematic of stepped centrifugation protocol for pelleting CPP and MBP from human serum. Representative electron micrographs are shown. Scale bar=100nm. CPP, calciprotein particles; MBP, membrane-bound particles; RT, room temperature; TEM, transmission electron microscopy.

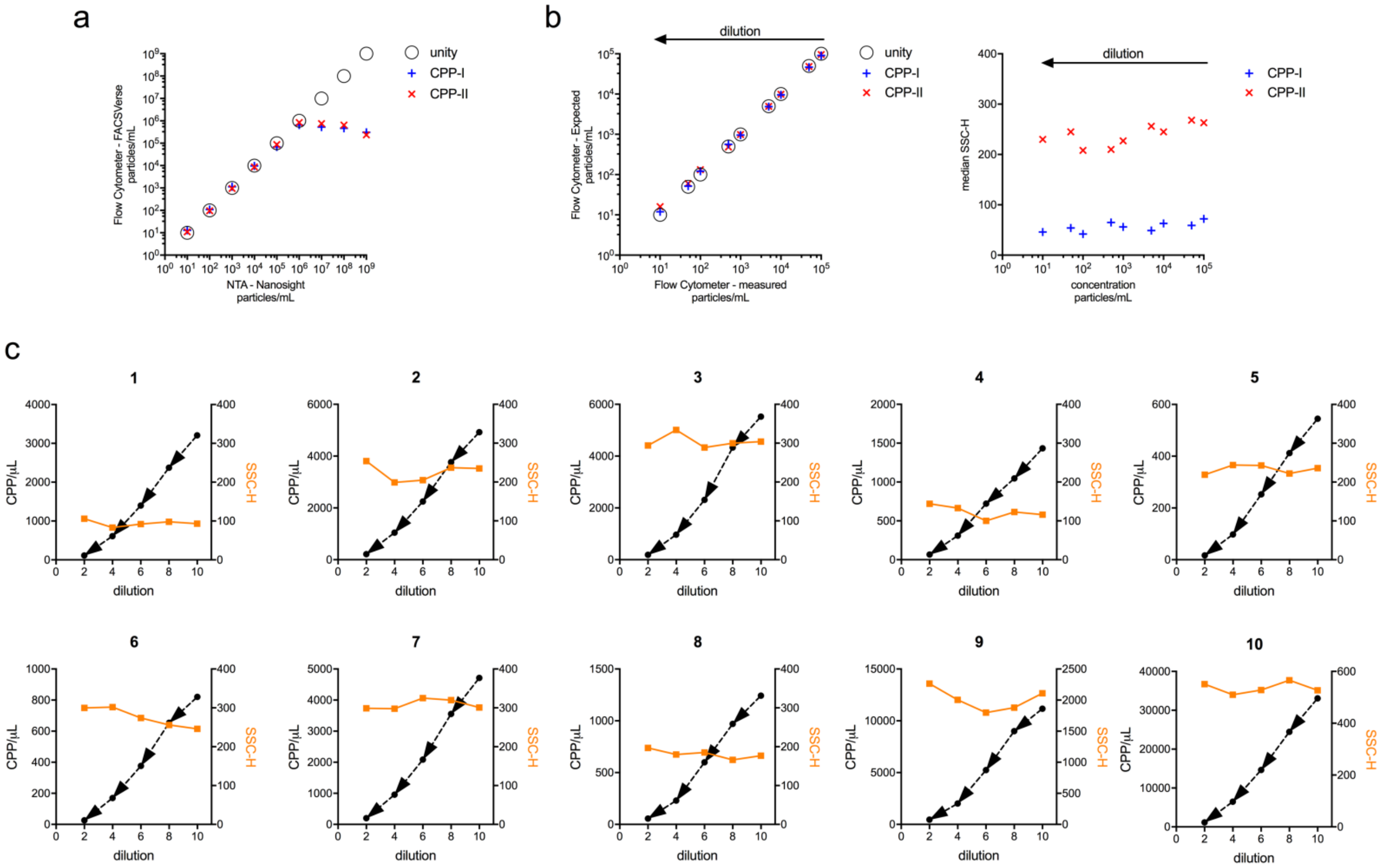

Fig S5. Assessment of the quantitative performance of the flow cytometry method. (A) Dilution studies of purified serum-derived CPP-I (blue +) and CPP-II (red X) with volumetric quantitation by flow cytometry and nanoparticle tracking analysis. Note departure from linearity at high concentrations. (B) Recovery studies of synthetic CPP-I and CPP-II showing agreement between expected and measured concentrations with dilution below 10^5 particles/mL. Note stability of SSC-H to dilution. (C) Dilution studies using uraemic human serum samples (n=10) showing near-linear reduction in particle counts (black) with stable SSC-H readout (orange). CPP, calciprotein particles; SSC, side scatter.

a

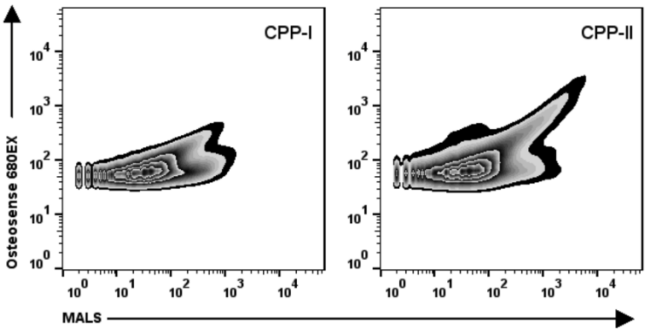

b

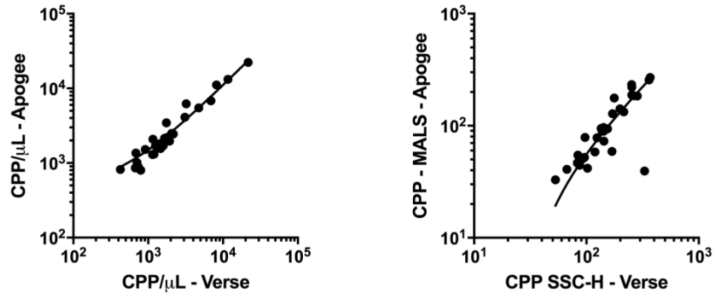

	Apogee
**Best-fit values ± SE**	
**Slope**	1.067 ± 0.03222
**Y-intercept**	431.3 ± 166.9
**X-intercept**	-404.1
**1/slope**	0.937
**95% Confidence Intervals**	
**Slope**	1.001 to 1.133
**Y-intercept**	89.33 to 773.3

	Apogee
**Best-fit values ± SE**	
**Slope**	0.771 ± 0.1035
**Y-intercept**	-21.52 ± 19.92
**X-intercept**	27.91
**1/slope**	1.297
**95% Confidence Intervals**	
**Slope**	0.559 to 0.983
**Y-intercept**	-62.33 to 19.3

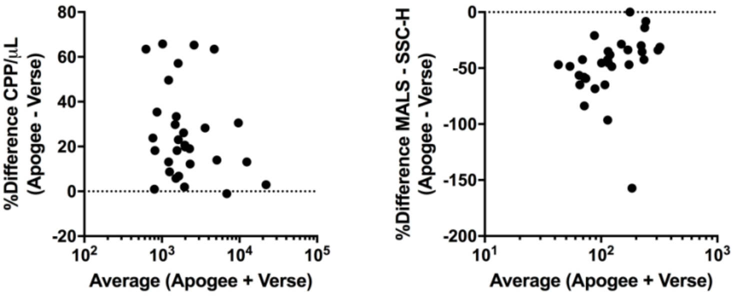

**Bias**	25.65
**SD of bias**	20.43
**95% Limits of Agreement**	
**From**	-14.39
**To**	65.69

**Bias**	-47.59
**SD of bias**	29.08
**95% Limits of Agreement**	
**From**	-104.6
**To**	9.404

Fig S6. Comparison of CPP characterisation and quantitation using BD FACSVerse and Apogee A50 Microflow cytometers. (A) Representative plots of OsteoSense 680EX positive serum-derived CPP-I and CPP-II and MALS on the Apogee microflow cytometer, both with fluorescence threshold triggering. (B) Deming regression analysis and Bland-Altman bias plots comparing total CPP quantitation and scatter signal intensities using the BD FACSVerse and Apogee. Summary statistics and 95% limits of agreement tabulated. Note linear agreement (slopes close to 1.0) between instruments, but slight negative bias for CPP/µL, and positive bias for scatter, using the FACSVerse relative to the Apogee A50. CPP, calciprotein particles; MALS, medium-angle light scatter; SSC, side scatter.

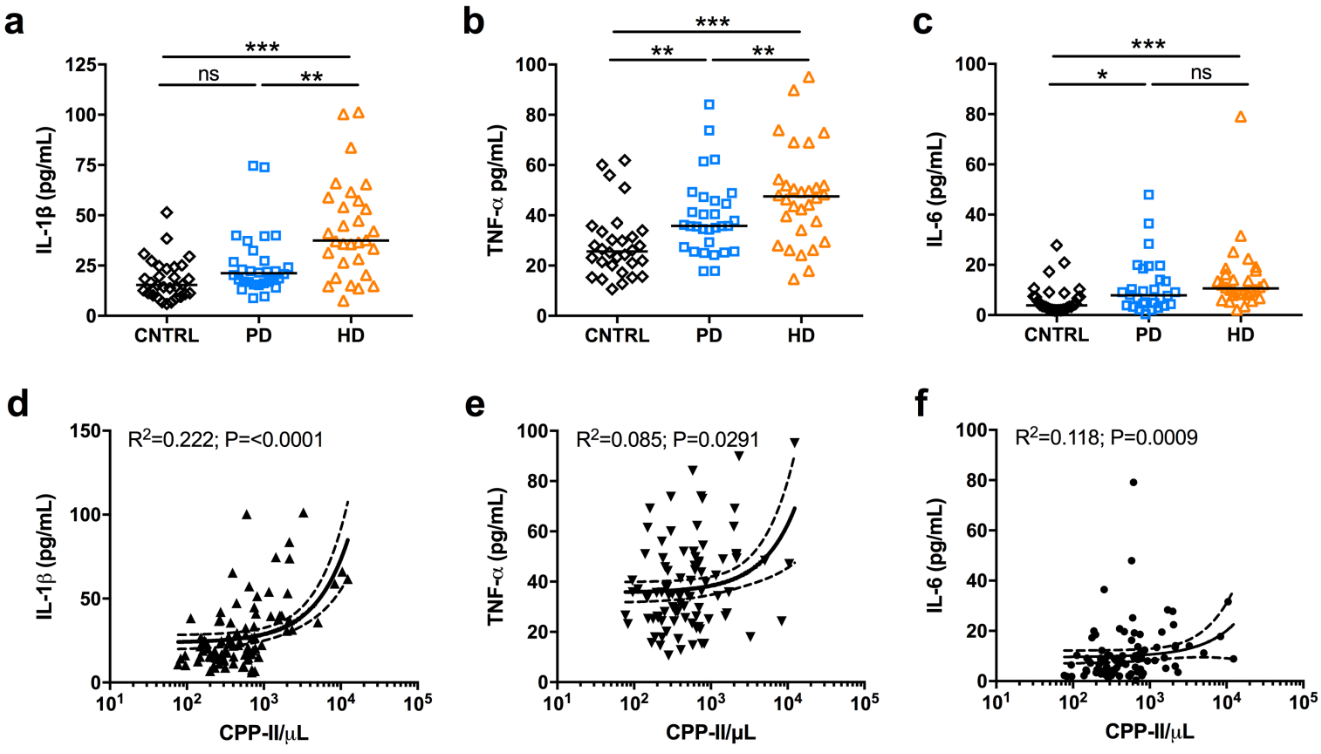

Fig S7. Association of serum CPP-II with inflammatory cytokine levels. (A-C) Differences in serum (A) IL-1β, (B) TNF-α and (C) IL-6 concentration between control (black diamonds), PD (blue squares) and HD (orange triangles) groups. Line=median value. (D-F) Non-linear regression analyses of CPP-II/μL and serum inflammatory cytokine concentrations. (A-C) P-values for one-way ANOVA with Bonferroni correction or Kruskal-Wallis test with Dunn's post-test for multiple comparisons denoted as *P<0.05, **P<0.01, ***P<0.001. For non-linear regression analyses (D-F), regression line (solid) with 95% confidence intervals (dashed) are shown. CNTRL, control; CPP, calciprotein particles; IL, interleukin; MBP, membrane-bound particles; HD, haemodialysis; PD, peritoneal dialysis; TNF, tumour necrosis factor.

serum - adenine-treated/HPD - 10 wks

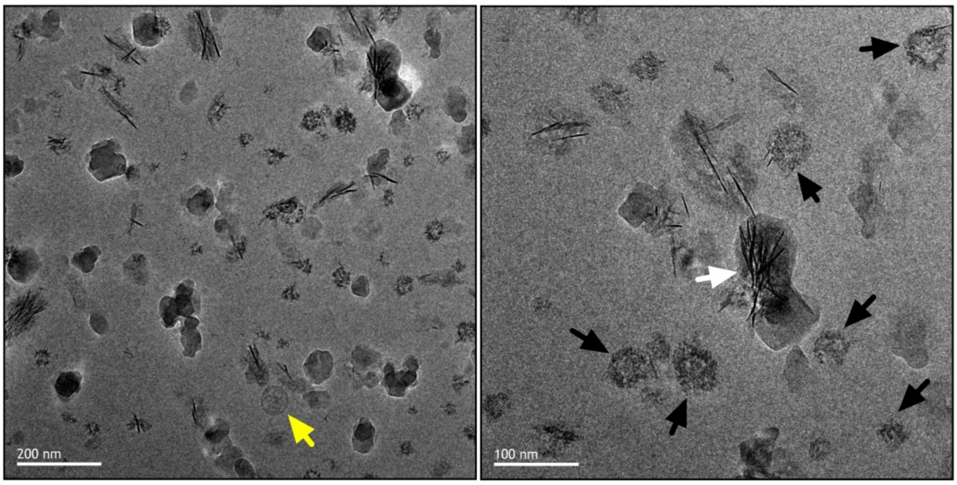

Fig S8. Microscopic analysis of mineral-containing nanoparticles in AIRF rat serum. Representative cryogenic transmission electron micrographs of CPP isolated by high-speed centrifugation (30,000 *g* for 2h at 4°C) from pooled uraemic rat serum (n=10) obtained at week 10. Scale bar =200nm. Higher magnification image shown alongside, scale bar=100nm. Yellow arrow denotes solitary vesicular structure. Black arrows indicate particles with CPP-I-like morphology. White arrow indicates particles with CPP-II-like structure.

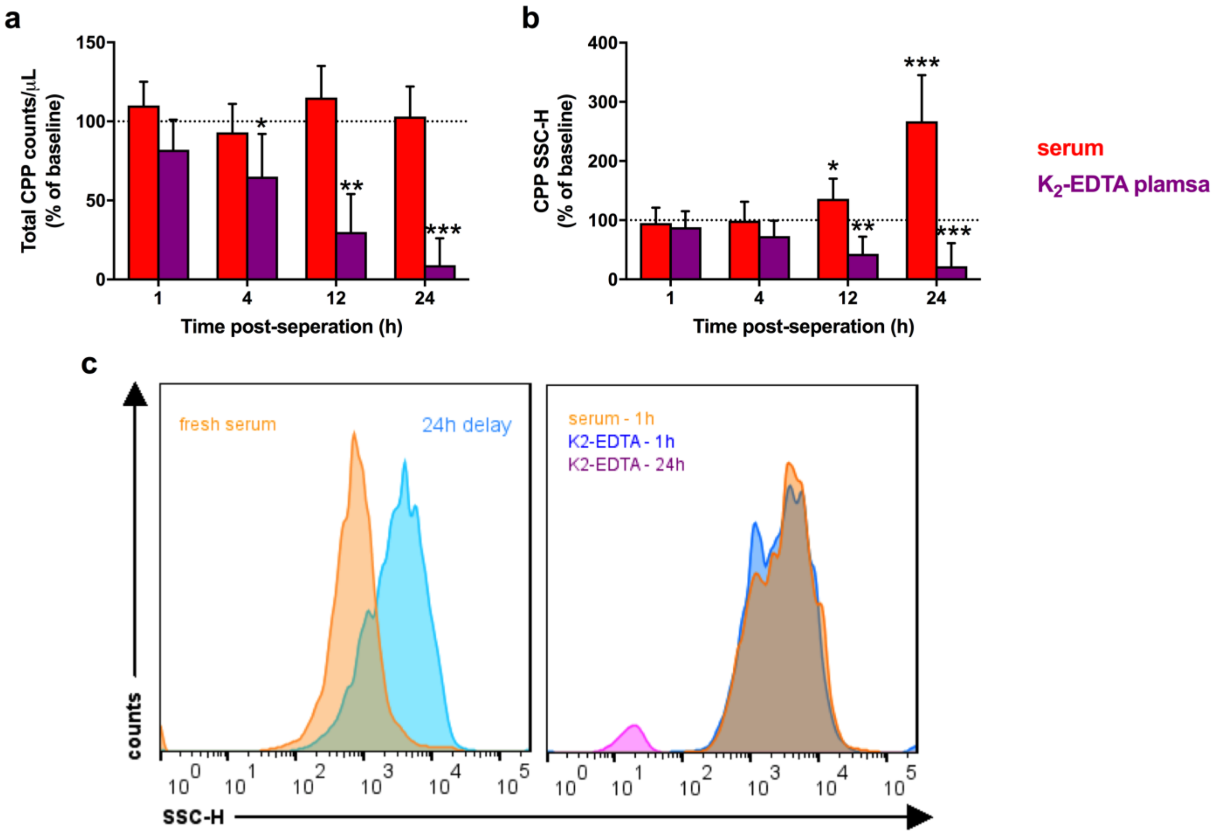

Fig S9. Sample stability studies. Time and sample type-dependent stability of (A) CPP total counts and (B) CPP SSC-H intensity in HD serum/plasma (n=4) after separation from cells. Data are expressed as percentage of values determined for samples processed immediately after separation of blood. (C) Representative histograms showing time- and sample-type dependent changes in SSC-H readings. P-values for two-way ANOVA with Dunnetts multiple comparisons test denoted as *P<0.05, **P<0.01, ***P<0.001. CPP, calciprotein particles; side scatter, SSC.

S1 Table. Medication use according to study subgroup.

Medication	Control (n=40)	PD (n=40)	HD (n=40)
nutritional vitamin D			
n (%)	-	21 (53)	29 (73)
dosage (μg/day)	-	1250 ± 355	3750 ± 2257
active vitamin D sterols			
n (%)	-	4 (10)	20 (50)
cinacalcet			
n (%)	-	5 (13)	8 (20)
dosage (mg/day)	-	50 ± 15	60 ± 26
calcium carbonate			
n (%)	-	12 (30)	32 (80)
dosage (mg Ca/day)	-	2130 ± 710	2258 ± 1455
sevelamer carbonate			
n (%)	-	8 (20)	14 (35)
dosage (mg/day)	-	3500 ± 1225	3967 ± 1076
lanthanum carbonate			
n (%)	-	5 (14)	17 (43)
dosage (mg/day)	-	2000 ± 1415	3725 ± 975
aluminium hydroxide			
n (%)	-	0	1 (3)
dosage (mg/day)	-	-	1800
no. of binders	-	1.2 ± 0.5	1.9 ± 0.5
bisphosphonate			
n (%)	-	0	1 (3)
β-blockers			
n (%)	-	19 (48)	17 (43)
Ca-channel blockers			
n (%)	-	18 (45)	19 (48)
ACEI/ARBs			
n (%)	-	17 (43)	14 (35)
diuretics			
n (%)	-	19 (48)	17 (43)
proton pump inhibitors			
n (%)	-	18 (45)	20 (50)
anti-platelet agents			
n (%)	-	5 (13)	17 (43)
lipid-lowering therapy			
n (%)	-	18 (45)	13 (33)
erythropoietin			
n (%)	-	28 (70)	33 (83)
iron supplementation			
n (%)	-	21 (53)	24 (60)

Expressed as mean ± SD
Abbreviations: ACEi, angiotensin-converting enzyme inhibitor; ARB, Angiotensin II receptor blockers; HD, haemodialysis; PD, peritoneal dialysis

S2 Table. ApogeeMix bead characteristics and detection efficiencies.

Material	Refractive index (η)	Particle size (nm)	Fluorescence	Detection efficiency	
				SSC	FL
latex	1.59	110	green	0.6±0.3	0.8±0.2
silica	1.43	180	none	0.7±0.3	-
silica	1.43	240	none	0.9±0.2	-
silica	1.43	300	none	1.0±0.2	-
latex	1.59	500	green	1.0±0.2	1.1±0.2
silica	1.43	590	none	0.9±0.3	-
silica	1.43	880	none	1.0±0.1	-
silica	1.43	1300	none	1.0±0.2	-

Detection efficiency is defined as the ratio of the measured and actual prepared concentration

FL, fluorescence; SSC, side scatter
